# Toward an Understanding of SEI Formation and Lithium Plating on Copper in Anode-Free Batteries

**DOI:** 10.1021/acs.jpcc.1c03877

**Published:** 2021-07-27

**Authors:** Svetlana Menkin, Christopher A. O’Keefe, Anna B. Gunnarsdóttir, Sunita Dey, Federico M. Pesci, Zonghao Shen, Ainara Aguadero, Clare P. Grey

**Affiliations:** † Department of Chemistry, University of Cambridge, Lensfield Road, Cambridge CB2 1EW, U.K.; ‡ Department of Materials, Imperial College London, Royal School of Mines, London SW7 2AZ, U.K.

## Abstract

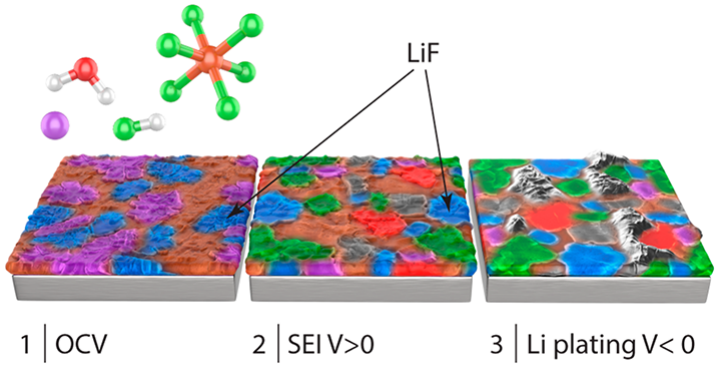

“Anode-free” batteries present a significant advantage due to their substantially higher energy density and ease of assembly in a dry air atmosphere. However, issues involving lithium dendrite growth and low cycling Coulombic efficiencies during operation remain to be solved. Solid electrolyte interphase (SEI) formation on Cu and its effect on Li plating are studied here to understand the interplay between the Cu current collector surface chemistry and plated Li morphology. A native interphase layer (N-SEI) on the Cu current collector was observed with solid-state nuclear magnetic resonance spectroscopy (ssNMR) and electrochemical impedance spectroscopy (EIS). Cyclic voltammetry (CV) and time-of-flight secondary ion mass spectrometry (ToF-SIMS) studies showed that the nature of the N-SEI is affected by the copper interface composition. An X-ray photoelectron spectroscopy (XPS) study identified a relationship between the applied voltage and SEI composition. In addition to the typical SEI components, the SEI contains copper oxides (Cu_*x*_O) and their reduction reaction products. Parasitic electrochemical reactions were observed via *in situ* NMR measurements of Li plating efficiency. Scanning electron microscopy (SEM) studies revealed a correlation between the morphology of the plated Li and the SEI homogeneity, current density, and rest time in the electrolyte before plating. Via ToF-SIMS, we found that the preferential plating of Li on Cu is governed by the distribution of ionically conducting rather than electronic conducting compounds. The results together suggest strategies for mitigating dendrite formation by current collector pretreatment and controlled SEI formation during the first battery charge.

## Introduction

The search for higher energy density rechargeable lithium (Li) batteries has created a renaissance of interest in the Li metal anode. However, potential safety issues due to Li dendrite growth and low cycling Coulombic efficiency are delaying the practical implementation of lithium metal batteries (LMBs).^1−3^ The use of an excessively thick layer of Li metal in full battery cells decreases the practical energy density^4^ and increases safety concerns. “Anode-free” or “Li-free” batteries aim to address these challenges, being composed of a Li-ion cathode and a copper (Cu) current collector, the cathode comprising the Li source. A thin layer of Li metal is then electroplated on the current collector during charging.^2^ These types of batteries present a significant advantage due to their higher energy density and ease of assembly in a dry air atmosphere.

The solid electrolyte interphase (SEI) is the key factor that determines the performance and safety of LMBs. It serves as an interphase between the metal and the electrolyte and has the properties of a composite solid electrolyte.^5,6^ It has been widely demonstrated in the literature that nonhomogeneous SEI compositions and morphologies initiate uneven plating and stripping of Li metal anodes, resulting in dendritic short circuits and low Coulombic efficiencies.^4,7−9^

The existing studies of the SEI formed on Cu suggest a different SEI chemical composition compared to the SEI formed on Li metal anodes.^2,10−13^ Chemical species that have been reported in the SEI on Cu include lithium carbide (Li_2_C_2_), lithium carbonate (Li_2_CO_3_), CuF_2_, Cu_*x*_O, Li_2_O, OH^–^ (or alkoxide), LiF, poly(ethylene glycol), and Cu nanoparticles.^3,14^ Interestingly, LiF is formed on Cu as a result of electrocatalytic reduction of HF at 2 V on the Cu surface, while the same reaction occurs at lower potentials on the traditional graphite anode (∼0.25 V).^11,15^

While Li plating and the SEI formation on Cu have been extensively studied,^11,16−19^ the role that the current collector plays has yet to be fully established. This study looks at the surface chemistry of the Cu current collector and its effect on the properties of the Li anode. The main objectives are to assign the chemical and electrochemical reaction products to specific processes, to quantify the reversibly plated and electrically isolated Li deposits (typically known as “dead Li”), to detect parasitic and SEI formation reactions, and to explain the uneven morphology of the plated Li. A study of these processes is required so as to understand the mechanisms for plating and stripping of Li metal on Cu and for the development of high-energy, long-life, and safe “anode-free” batteries.

The native surface oxide film on alkali metal anodes is commonly termed a native SEI (N-SEI). Here the definition of N-SEI is expanded to the surface passivation layer on the Cu current collector prior to the application of current or potential step, beyond that already present on the Cu (but it will vary depending on how Cu is stored and chemically treated), due to the spontaneous reactions that occur upon immersion in an electrolyte.^20^ The SEI on the Cu current collector further evolves when current is passed, prior to the onset of Li plating, and this is termed the electrochemical SEI (e-SEI) in this study.

This paper reports the effect of the surface chemistry of the Cu current collector on the chemical composition of the SEI and the plating of Li metal. The Results section will follow the SEI formation process first on Cu and then during Li plating, namely N-SEI formation and e-SEI formation, and will investigate Li plating and dead Li formation.

A typical preparation procedure for Cu current collectors in the literature is the pretreatment by dilute hydrochloric or concentrated acetic acids^21,22^ (the current collectors being termed d-HCl-Cu and c-AcH-Cu, respectively). The d-HCl surface treatment and the corresponding SEI that forms were here compared to the SEI on c-AcH-Cu, the latter being chosen because the c-AcH surface treatments have been reported to give rise to thicker and more homogeneous Cu oxide surface layers.^23^

We show with ssNMR and EIS that the native interphase (N-SEI) is formed instantaneously on Cu current collectors upon their immersion in a LiPF_6_-based electrolyte. Parasitic electrochemical reactions were observed in the course of the first five cycles in Cu–LiFePO_4_ (LFP) cells via *in situ* NMR,^24^ resulting in lower Li plating efficiency on Cu than that measured electrochemically. The morphology of the plated Li and efficiency of the Li plating–stripping process were shown to depend on the homogeneity of the Cu oxide layer and the aging N-SEI on Cu. Preferential plating of Li, dictated by the local composition of the SEI and its ionic conductivity, was demonstrated by ToF-SIMS and XPS.

## Experimental Section

### Materials

The electrolyte used was 1 M LiPF_6_ in 1:1 v/v ethylene carbonate/dimethyl carbonate (EC/DMC; Sigma-Aldrich, battery grade, LP30). The water content in the electrolyte was 10–30 ppm (measured with Karl Fischer titration, Metrohm). In all the coin cells, precut Li metal disks (LTS research, 99.95%) were used. The materials were stored and handled in an Ar atmosphere glovebox (O_2_, H_2_O < 1 ppm, MBraun). Cu metal foil (MTI) was used as a substrate for Li plating. Cu metal flakes (average particle size of 0.4 mm, Aldrich), CuO (<50 nm, Aldrich), and Cu_2_O (<350 nm, Aldrich) were used as Cu/Cu_*x*_O substrates for N-SEI samples for ssNMR experiments. Acetic acid (Fischer Chemical; Lab reagent grade, glacial) was used as received. Hydrochloric acid (Honeywell, Fluka, (07102)) was diluted to 1.1 M in deionized water. Polypropylene–polyethylene (PPPE) (Celgard) and glass fiber (Whatman) separators were dried under vacuum at 40 and 100 °C, respectively.

### Current Collector Pretreatment

Cu metal disks and Cu metal flakes were soaked in either concentrated acetic acid (denoted c-AcH-Cu) or dilute hydrochloric acid (denoted d-HCl-Cu) for 10 min for surface oxide removal. The d-HCl-treated Cu was afterward rinsed with acetone. Following a previously reported surface treatment, the acetic acid on c-AcH-Cu was removed with a dry nitrogen flow.^23^ The disks were then dried at 100 °C under vacuum overnight. Because both hydrochloric dilute acid and acetic acid do not dissolve metal copper, the trenched morphology of the Cu foil remains after the treatment.

### Cell Fabrication

Symmetric cells, composed of two pretreated Cu current collectors (thickness 25 μm), and Cu–Li half-cells were assembled in coin cells (Cambridge Energy Solutions) with three stainless steel disks (thickness 0.5 mm) and 75 μL of LP30 electrolyte.

The *in situ* NMR cells were assembled in a capsule cell (NMR Service GmbH) made out of poly(ether ether ketone) (PEEK) as described previously.^25^ LiFePO_4_ (LFP) cathodes (MTI) were cast on carbon-coated Al foil. The electrodes (LFP and Cu) were cut into ∼0.4 cm^2^ rectangular electrodes. Slightly larger rectangular PPPE and glass fiber separators were sandwiched between the LFP cathode and Cu, with the PPPE facing the Cu, by using 100 μL of LP30 electrolyte.

Three-electrode cells, composed of a Cu current collector working electrode (WE), a Li counter electrode (CE), and a Li quasi-reference electrode (RE), were assembled in three-electrode electrochemical cells (RHD instruments) by using 600 μL of LP30 electrolyte.

### Electrochemistry

**Figure 1:**
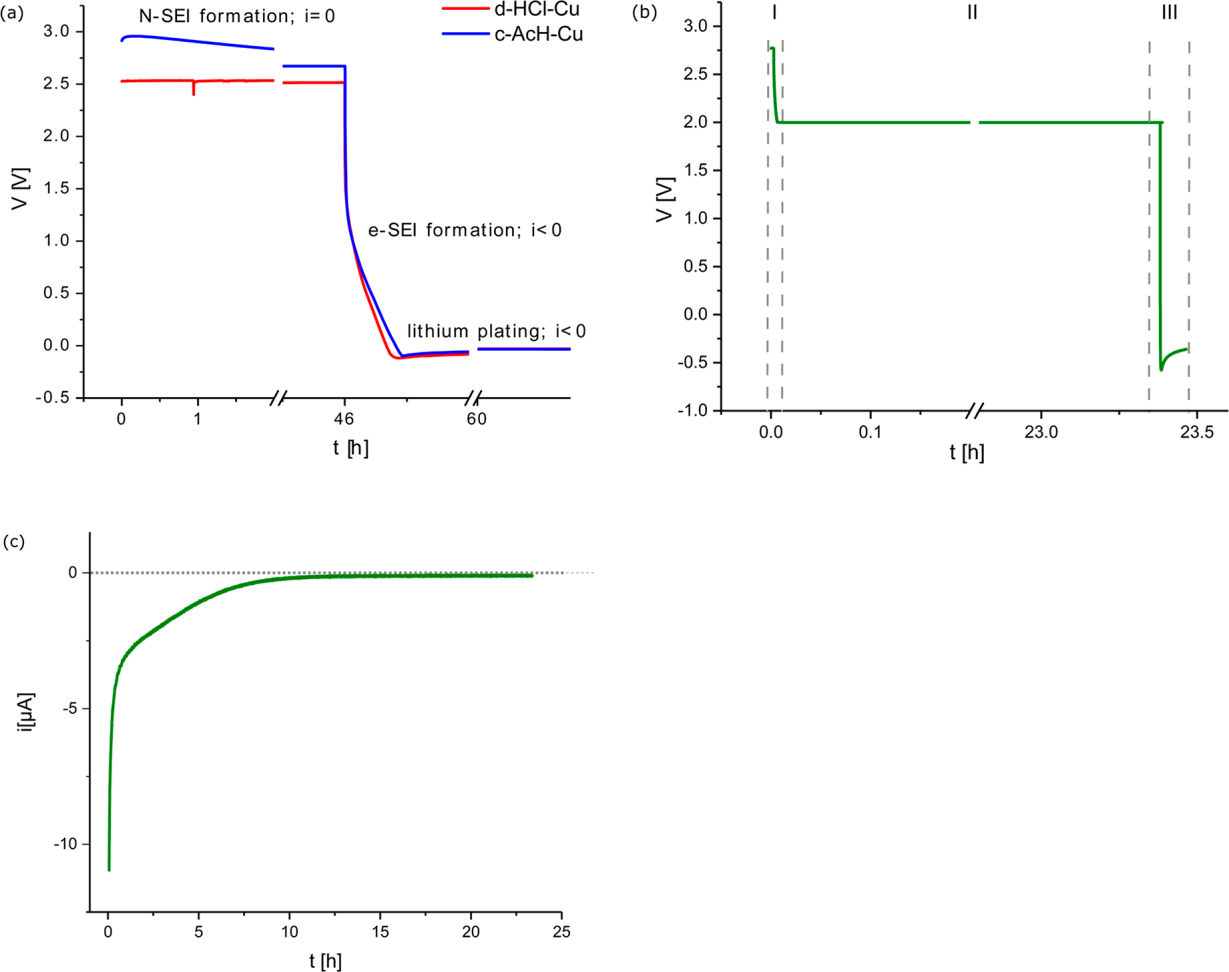
(a) A typical voltage response, after cell assembly, for Cu-Li half cells made with the two Cu surface treatments, d- HCl and c-AcH. The rest period at OCV is followed by galvanostatic SEI formation and Li deposition on Cu using a 0.03 mA cm^–2^ current density (normalized to the Cu WE area). This procedure was used to prepare the samples for SEM and XPS. (b) A typical voltage response during SEI formation on Cu using 0.03 mA cm^–2^ current density, between OCV and 2 V (I), followed by eSEI_2V_ formation during a voltage hold at 2 V (II) and galvanostatic Li deposition using 1.2 mA cm^–2^ current density (III). (c) A typical current response during the constant voltage hold at 2 V (denoted as stage II in (b)). The data presented in (b) and (c) are measured on c-AcH-Cu. The profile depicted in (b) was used to prepare the samples for ToF-SIMS.

N-SEI was formed on Cu after the assembly of a Li–Cu coin cell during the cell storage prior to Li plating. SEI formed on Cu at constant voltage *X*V is denoted eSEI_*X*V_, for example, eSEI_0.1V_ for SEI formed at 0.1 V. Li plating was performed at a constant current density in the range 0.03–1.2 mA cm^–2^ after either a rest time at OCV of 0–12 h or a constant voltage hold (typical deposition curves are depicted in Figures 1a and 1b, respectively). For the ToF-SIMS and XPS studies of Li preferential plating, thin (0.1 mAh cm^–2^) Li nucleation layers were plated on the Cu at 1.2 mA cm^–2^ current density. Potentiostatic electrochemical impedance spectroscopy (PEIS) was conducted in the 1 MHz–1 Hz frequency range with an amplitude of 10 mV (Biologic Instruments, VSP 300 potentiostat).

For the *in situ* NMR experiments, galvanostatic cycling was performed on LFP-Cu cells by using a current density of 0.1 mA cm^–2^ in the first cycle followed by 0.5 mA cm^–2^ in subsequent cycles. A cutoff capacity of 1 mAh cm^–2^ was used during charge (plating) and a cutoff voltage of 2.8 V during discharge (stripping). Note that the LFP cathode was not fully delithiated; the areal capacity of the LFP cathode is roughly 2.3 mAh cm^–2^.

### Sample Preparation and Transfer

For the preparation of samples for the ssNMR study of the N-SEI, Cu metal flakes or Cu oxide powders were soaked in the electrolyte either with or without the presence of Li metal, sealed in a coin cell, and left for variable periods of times ranging from 1 to 96 h.

To study the e-SEI by ssNMR, the samples were prepared by the procedure for e-SEI reported above, followed by Li plating at 1.2 mA cm^–2^ and 0.5 mAh cm^–2^ capacity. The plated Li metal was then scraped gently from the Cu surface, and the samples were mixed with ground KBr (dried under vacuum at 200 °C for 48 h) and packed into 2.5 mm outer diameter zirconia rotors. For the SEM, XPS, and ToF-SIMS, the samples (deposited on Cu disks) were mounted in an airtight transfer module stage. The samples were dried under vacuum in the glovebox antechamber for ∼15 min prior to sample packing.

### NMR

The ssNMR experiments were conducted on a Bruker Avance IIIHD 500 MHz spectrometer using a 2.5 mm double channel Bruker MAS probe. ^19^F and ^7^Li chemical shifts were referenced externally by using LiF as a secondary reference (δ_iso_(^19^F) = −204 ppm and δ_iso_(^7^Li) = −1 ppm). Magic angle spinning (MAS) speeds of up to 30 kHz were used. The ^19^F and ^7^Li ssNMR was conducted by using a rotor-synchronized Hahn echo pulse experiment with optimized 90° pulse lengths of 2.7 μs (^19^F) and 3.3 μs (^7^Li), with 5 and 1 s recycle delays for ^19^F and ^7^Li, respectively.

The *in situ* NMR experiments were conducted on a Bruker Avance 300 MHz spectrometer (the respective Larmor frequency for ^7^Li being 116.6 MHz). The spectra were recorded by using an *in situ* ATMC probe (NMR Service GmbH) which contains leads that connect to a battery positioned inside a solenoidal Ag-coated Cu coil and to the potentiostat for battery cycling. The chemical shift of ^7^Li was referenced to 1 M LiCl in water at 0 ppm. Single-pulse experiments were used to collect the NMR data, with a recycle delay of 1 s (*T*_1_ for Li metal ∼100 ms), and 128 transients were recorded. This resulted in an experimental time of about 2.5 min. The spectra were processed in Bruker Topspin software by using the automatic phase and baseline correction. Further data processing was done in R.

### SEM

SEM images were taken with a Tescan MIRA3 FEG-SEM instrument at an acceleration voltage of 5.0 kV. The electrodes were not rinsed with solvent but mounted onto the SEM stage of the airtight transfer module (Kammrath & Weiss, type CT0) and transferred into the SEM chamber under an inert Ar atmosphere from the glovebox.

### XPS

A Thermo Scientific K-Alpha XPS system with a monochromated microfocused Al Kα X-ray source (*h*ν of 1468.7 eV) was used at a power of 72 W (12 kV × 6 mA). Samples were adhered to an airtight transfer chamber (Thermo Fisher Scientific, sample area 60 × 60 mm^2^) equipped with X-ray-transparent windows and were brought down to a measurement pressure of 10^–8^ mbar. The spot size of measurement was 200 μm^2^ with a step size of 5 μm. Along with the high-resolution survey scan, element specific energy scans were recorded (step size 0.1 eV/min). Spectra were recorded after 10 and 20 nm sputtering (sputtering rate of 0.5 nm/s) with an EX06 monatomic Ar ion source (MAGCIS) of energy 2 keV. Data processing, including background correction (Shirley function) and peak fitting (Gaussian–Lorentzian functions), was performed by using the CasaXPS software (ver. 2.3.15). The C 1s photoelectron peak position of 284.8 eV was used as an internal reference.

### Secondary Ion Mass Spectrometry (SIMS)

SIMS analysis was performed on a time-of-flight (ToF) SIMS 5 spectrometer (ION-TOF GmbH, Münster, Germany). The measurements were conducted in the burst alignment mode (BAM) for better lateral resolution of images with a Bi^+^ primary beam (25 keV) and a Cs^+^ sputtering beam (500 eV) over an area of 250 μm × 250 μm (sputtering area 500 μm × 500 μm). Additionally, for data analysis, the intensities of the species for different samples were point-to-point normalized to the total counts, and 3D reconstruction was also performed by using Surfacelab 6.0 software to obtain the distribution of different species.

## Results

### Electrochemistry

To study the effect of the Cu oxide layer composition on Li metal plating, two different oxide removal procedures^23^ were used to remove the inhomogeneous oxide–hydroxide layer on the untreated Cu metal, as described in the Experimental Section. A typical Li deposition curve for galvanostatic plating for Cu–Li half-cells is shown in Figure 1a for the two Cu surface treatments d-HCl-Cu and c-AcH-Cu. The procedure used in Figure 1a was chosen to resemble a realistic first formation charge of the anode-free Li battery. This procedure was used to prepare the samples for SEM and XPS analysis with the aim of exploring Li morphology and the SEI composition on Li-plated Cu.

To study the composition of the SEI that forms at specific potentials (eSEI_*X*V_) with XPS, constant current was applied until the desired voltage was reached; this was followed by a constant voltage hold (Figure 1b, stages I and II). To investigate whether there is preferential Li plating, the constant voltage holds were followed by Li plating at a relatively high current (1.2 mA cm^–2^) (Figure 1b, stage III). The high Li plating current was used to maximize metal plating and minimize SEI formation at lower voltages.^12,26^ During the voltage hold, the current exponentially decays as the reduction reactions appropriate for the applied voltage occur (Figure 1c). The voltage was held constant until the current signal decayed to neglectable values (Figures 1b and 1c). The voltage holds were chosen to fit the typical potentials for Cu oxide reduction and lithiation^27−33^ and the characteristic SEI formation potentials in LiPF_6_-based electrolytes.^18,34^ These samples were then studied by using TOF-SIMS, as described later.

The OCV value in the Cu–Li half-cells prior to any applied current fluctuates around 2.8 ± 0.4 V for both surface treatments. The differences in the OCV are ascribed to variations in the copper oxide and copper fluoride layers on the Cu surface^33^ and the spontaneous formation of the N-SEI passivation layer. A wide distribution of OCV values is observed between coin cells, which indicates that the N-SEI formation processes are dynamic and sensitive to both surface chemistry and cell assembly.

**Figure 2:**
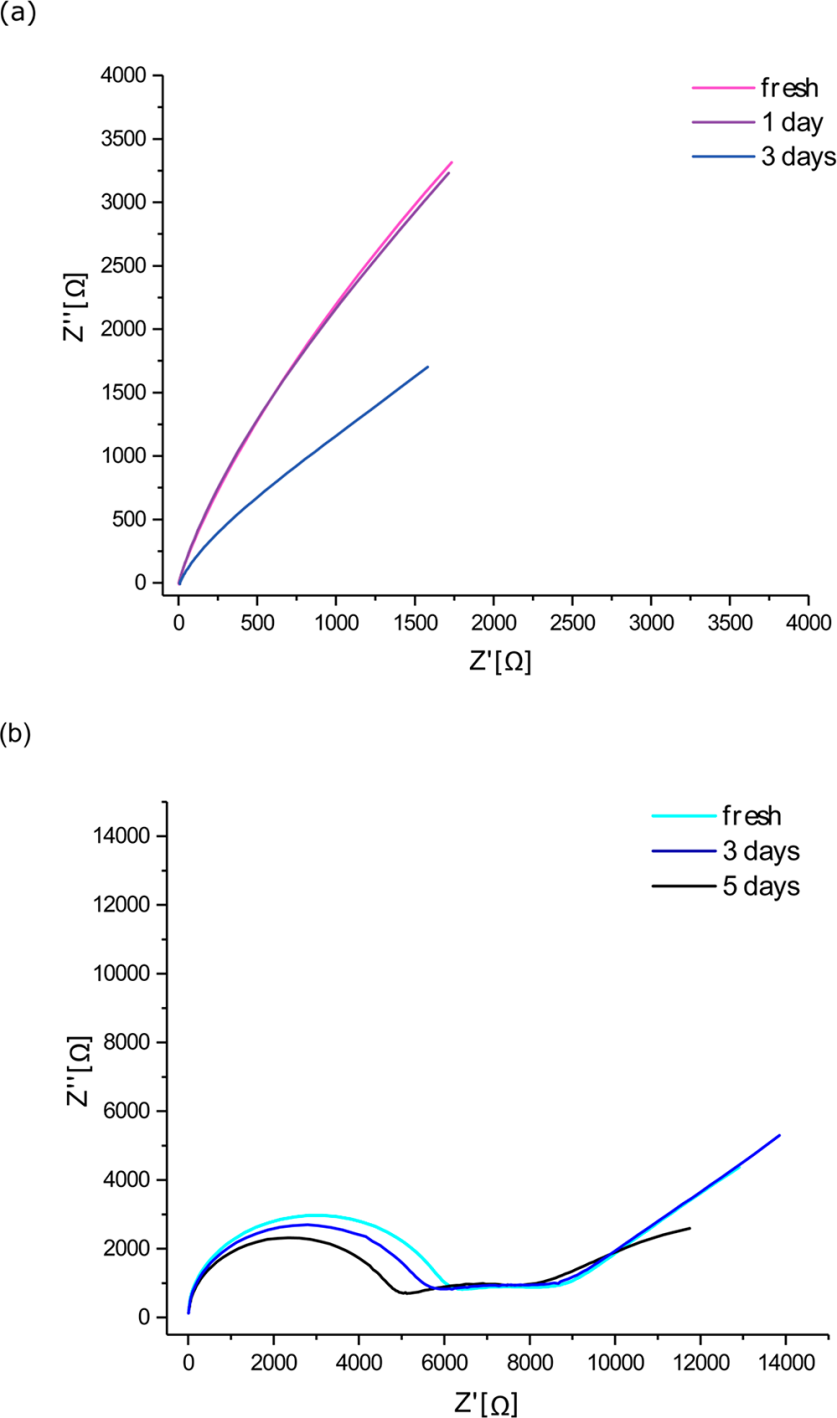
Nyquist plot of Cu–Cu symmetric cells of (a) d-HCl-Cu and (b) c-AcH-Cu foils performed with PEIS in the 1 MHz–1 Hz frequency range, with an amplitude of 10 mV around the OCV.

To explore the properties of the N-SEI, EIS was performed on Cu–Cu symmetric coin cells as a function of time, starting 1 h after cell assembly (Figure 2). The Nyquist plot of the symmetric d-HCl-Cu cell shows a typical response for a linear restricted diffusion toward a blocking electrode (Figure 2a).^35^ In contrast, the Nyquist plot for the symmetric c-AcH-Cu cell (Figure 2b) was fitted with Voigt-type equivalent circuit (which includes at least one resistor-capacitor (RC) unit), typical of a surface (passivating) film.^35^ This could either be due to more homogeneous passivation by Cu(II) oxide on the c-AcH-Cu, as demonstrated below by XPS, or the formation of a more dense N-SEI. After 5 days of rest, an additional RC component was needed to fit the impedance response, although the total impedance was not changed significantly (Figures S1 and S2, Table S1), suggesting an increase in the heterogeneity of the N-SEI after the prolonged rest time.

**Figure 3:**
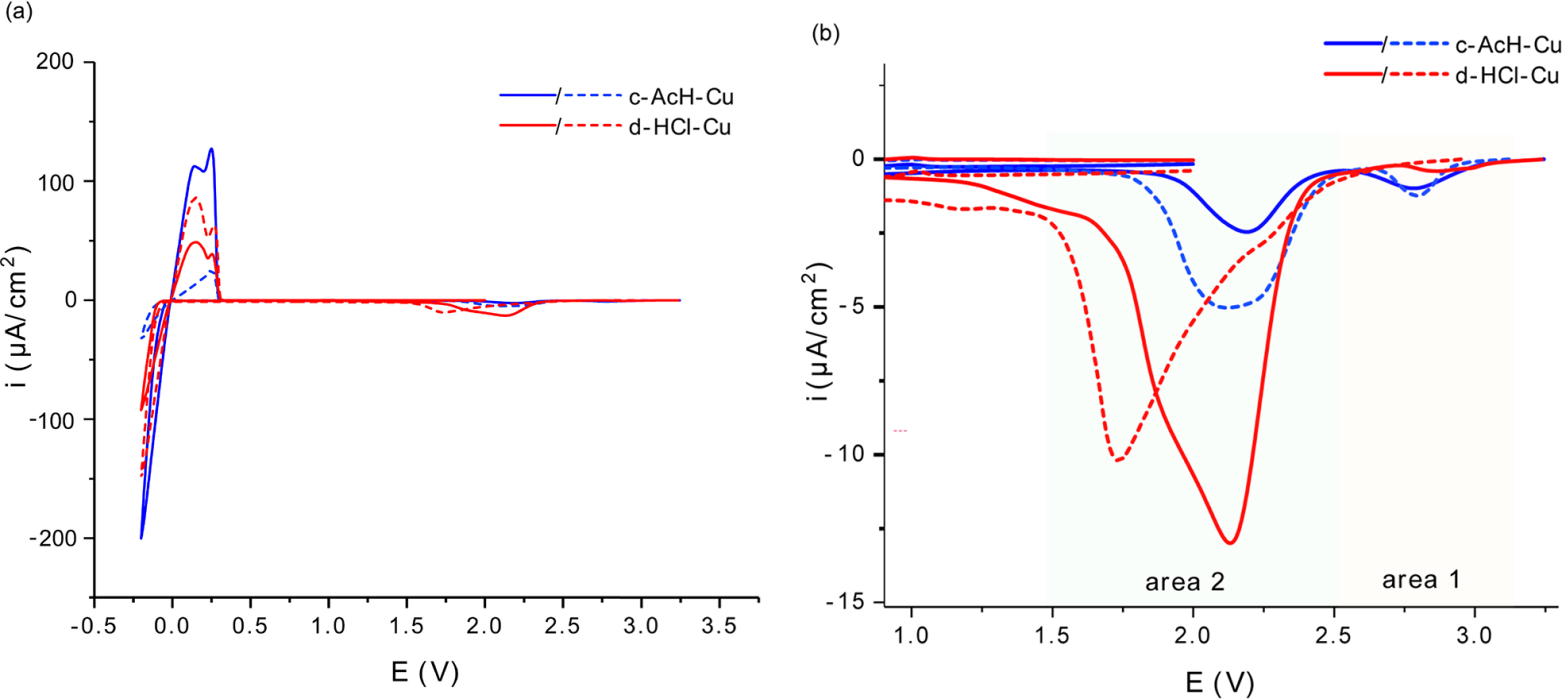
(a) First cycle CV of lithium plating on c-AcH-Cu (blue) and d-HCl-Cu (red) in three-electrode cell, Cu WE vs Li disk CE and RE, with scan rate 1 mV/s (full line: sample 1; dashed line: sample 2). (b) Expanded view of the CV in (a), indicating the higher potential ranges of interest. The voltage provided on the *x*-axis is the potential of WE vs RE.

Cyclic voltammograms (CV) of the first cycle of Li plating on c-AcH-Cu and d-HCl-Cu were measured in a three-electrode cell in the potential range of OCV to −0.2 V vs Li (Figure 3). The CV shows reduction peaks in three main voltage ranges. The weak peaks in the range of 2.5–3.1 V (area 1) are assigned to CuF_2_ reduction, which results in LiF and Cu formation by the following reaction:^27,36^
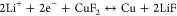
1Interestingly, the intensity of the peaks seen in area 1 is lower on d-HCl-Cu (Figure 3b, red). The peaks at around 2.3–1.3 V (area 2) are attributed to Cu oxide reduction, Cu_*x*_O lithiation, and LiF formation via the electrocatalytic route.^29,37^ The areas of the peaks around 2 V are larger for d-HCl-Cu (Figure 3b). The peaks at around 0.4 to −0.2 V (Figure 3a) are partially reversible and correspond to SEI formation, Li plating, and stripping. The partially reversible peaks around 0.4–1 V (as well as Li plating and stripping) appeared for the first ten cycles (see CV for cycles 2 and 10 in Figure S25). These peaks could be attributed to reversible SEI formation reactions, such as the reversible reduction of Li_2_CO_3_.^36,39^ The presence of the typical reduction products of Li_2_CO_3_ (Li_2_C_2_ and Li_2_O) in the SEI (observed by XPS, as described later) supports the assignment of these peaks to Li_2_CO_3_ reduction. Cu metal, which forms in the Cu oxide reduction, has been proposed to catalyze the reduction of Li_2_CO_3._^38,39^

### XPS

**Figure 4:**
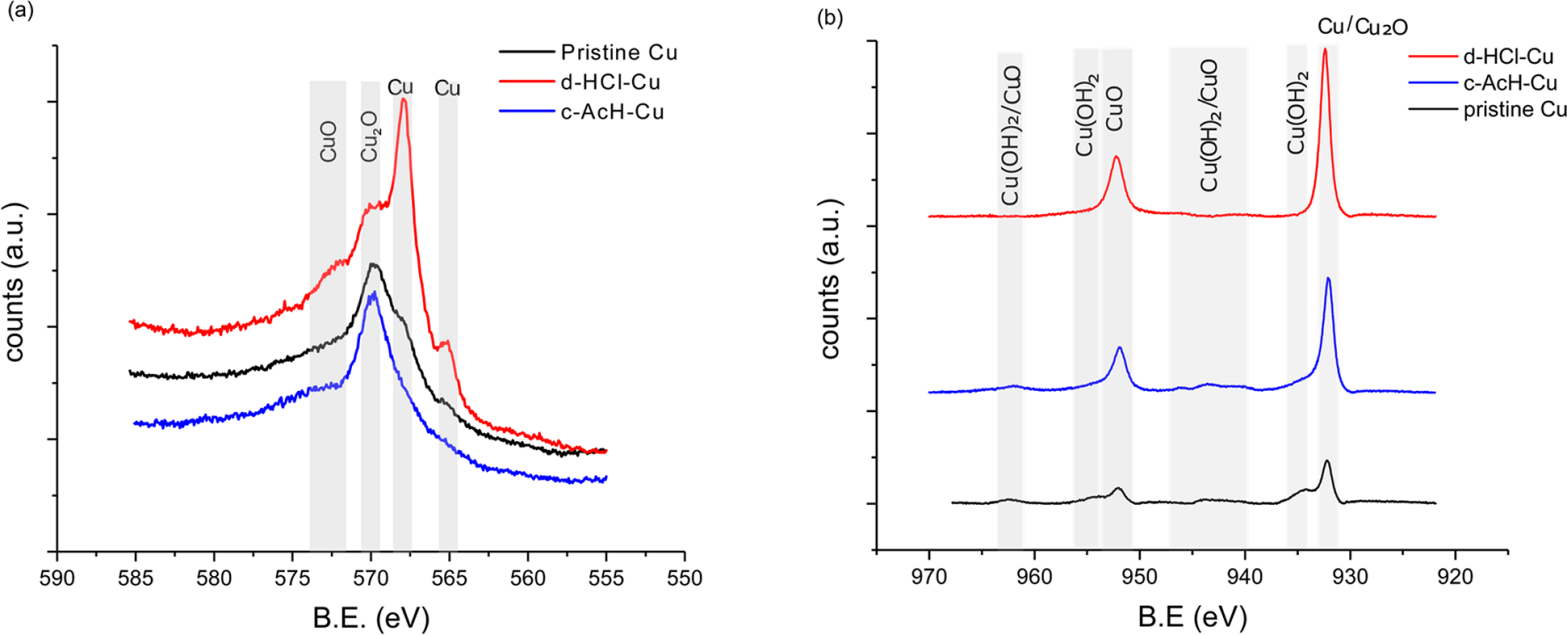
Auger spectra (a) and Cu 2p XPS spectra (b) of nontreated Cu (black), d-HCl-Cu (red), and c-AcH-Cu (blue).

The surface of d-HCl-Cu and c-AcH-Cu foils was studied by XPS (Figure 4). The Auger Cu metal peaks (565.0, 568.0, and 572.6 eV),^41,42^ which are only seen in the spectrum of d-HCl-Cu, indicate bare areas with thinner or no oxides and a more heterogeneous oxide layer on the d-HCl-Cu surface compared to both the c-AcH-Cu and the nontreated Cu metal (Figure 4a). The peaks at 931.0–932.4 eV (Cu 2p_3/2_) and 951.9–952.2 eV (Cu 2p_1/2_), observed in the Cu 2p spectra (Figure 4b) and the O 1s peak at 530.2 eV (Figure S13), are assigned to Cu metal and Cu(I) copper oxides. The shoulder observed in the Cu 2p spectra of c-AcH-Cu and the nontreated copper current collector at 934.0–935.0 eV, the minor shakeup feature at 941.0–945.0 eV, and the 531.0–531.2 eV peaks in the O 1s spectrum (Figure 4b and Figure S13) serve as an indication of the presence of Cu(II) compounds, likely Cu(II) (oxy)hydroxide.

XPS was then further used to study both the N-SEI and e-SEI formed on Cu. The XPS analysis of the N-SEI suggests that it is composed of Cu oxides (see the Supporting Information for detailed XPS analysis, Figures S13 and S14) and LiPF_6_ decomposition products, LiF and LiPO_*x*_F_*y*_ (Figure S16).

The e-SEI_1.4V_ (the SEI formed by a constant voltage hold at 1.4 V) is seen to be dominated by C–O species as evident by the intense XPS C 1s and O 1s peaks at 286.6 and 533.4 eV, respectively (Figures S16a and S16b). Sputtering of ∼10 nm resulted in C 1s C–C/C–H peak intensity (at around 284.4 eV), and additional at C 1s 289.7 eV and O 1s 532.5 eV (Figure S18). These findings could be attributed to the formation of short and medium length Poly(ethylene oxide)-like polymeric species on the surface and Li_2_CO_3_ in the inner layers of the e-SEI_1.4V_.^18,43,44^ Polymeric species were also observed on e-SEI_2V_ (see detailed analysis in the Supporting Information, Figures S16–S18).

The F 1s XPS spectrum of the e-SEI_1.4V_ shows that the signal attributed to LiF is significantly less dominant compared to that in the spectra of the N-SEI and e-SEI_2V_ (Figure S16c). However, sputtering on the e-SEI_1.4V_ (by roughly 20 nm) revealed a nearly 3-fold increase in the LiF:LiPF_6_ ratio in comparison to the surface (Figure S19 and Table S2). Thus, LiF is mostly found in the inner part of the interphase of the e-SEI_1.4V_, where the LiF formation most likely occurs during rest periods and at voltages above 1.4 V. This finding was supported by ToF-SIMS depth profiles of LiF on N-SEI and e-SEI formed at 2–0.1 V (Figure S27).

### NMR

**Figure 5:**
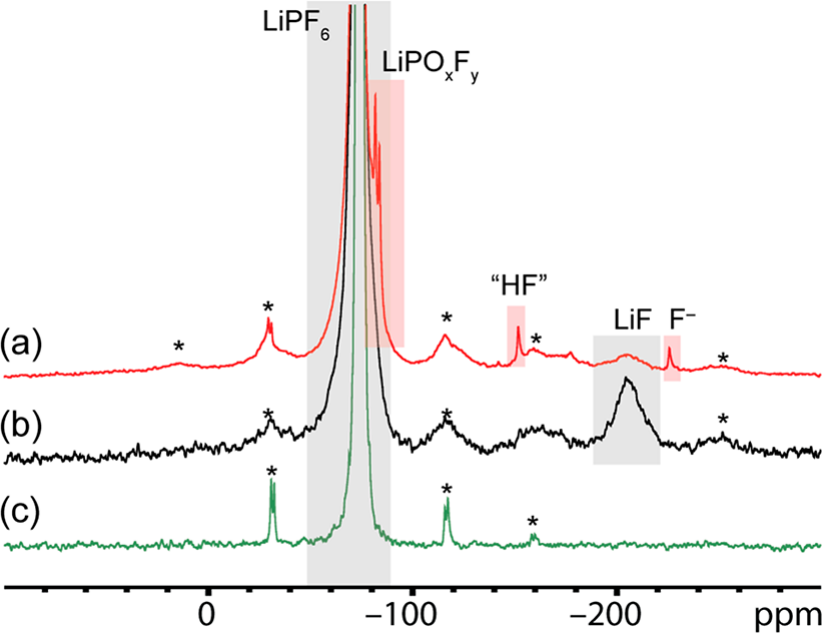
^19^F ssNMR spectra of (a) d-HCl-Cu flakes soaked in LP30 for 1 h, (b) Li metal soaked in LP30 for 1 h, and (c) 75 μL of LP30 mixed with KBr and dried. The spectra were acquired with a MAS frequency of 20 kHz. Spinning sidebands are denoted with asterisks.

The N-SEI and e-SEI on Cu were studied by ^19^F and ^7^Li ssNMR. To increase the signal-to-noise ratios in the ssNMR spectra and mimic the N-SEI, acid-treated Cu metal flakes were soaked in the LP30 electrolyte and then dried and packed into the MAS rotors. MAS of metallic samples is challenging as the rotation in a magnetic field induces eddy currents which results in unstable spinning. To alleviate this issue, the samples were diluted with dried KBr powder. In Figure 5, the ^19^F NMR spectra for N-SEI on Li metal and d-HCl-Cu soaked in LP30 electrolyte are compared to that of the dried LP30 electrolyte mixed with KBr. For the Li and Cu samples, two groups of resonances between −72 and −85 ppm and between −202 and −204 ppm were observed. The first group of resonances is assigned to LiPF_6_ salt and its decomposition products, and the second group is assigned to LiF.^45,46^ LiF was also observed in the ^19^F NMR spectra of Cu oxides (Cu_2_O and CuO) powders soaked in LP30 electrolyte (Figure S3).

As shown in Figure 5c, only signals corresponding to LiPF_6_ were observed for the solidified electrolyte in dried KBr, indicating that the generation of LiF results from the reaction of the electrolyte on the metals surface and is not due to decomposition of the bulk electrolyte or a reaction with the KBr. This indicates that the fluorine-containing N-SEI can form upon soaking the metal and oxides in an electrolyte without any applied potential. This reaction is likely triggered by water or hydroxides in the oxide passivating films.

The weak resonance at −225 ppm is unique to soaked Cu samples, that is, N-SEI on Cu (see Figure 5a and Figures S3–S5). There is no obvious assignment to this resonance: it is usually assigned to NaF; however, NaF was not seen in the ^23^Na ssNMR spectra of the same samples.

To compare the N-SEIs on d-HCl-Cu and c-AcH-Cu, c-AcH-Cu flakes were soaked in LP30 for 1–24 h (Figures S4 and S5) and studied with ssNMR. The sample soaked for 24 h had additional sharp resonances around −131, −142, −151, −170, and −184 ppm (Figure S5). This demonstrates that the formation of the N-SEI and the decomposition of the LiPF_6_ salt are a dynamic process that is highly dependent on the Cu surface chemistry and soaking time (see detailed discussion on ^19^F NMR of e-SEI_2V_ and e-SEI_1.4V_ in the Supporting Information, Figures S4–S10).

**Figure 6:**
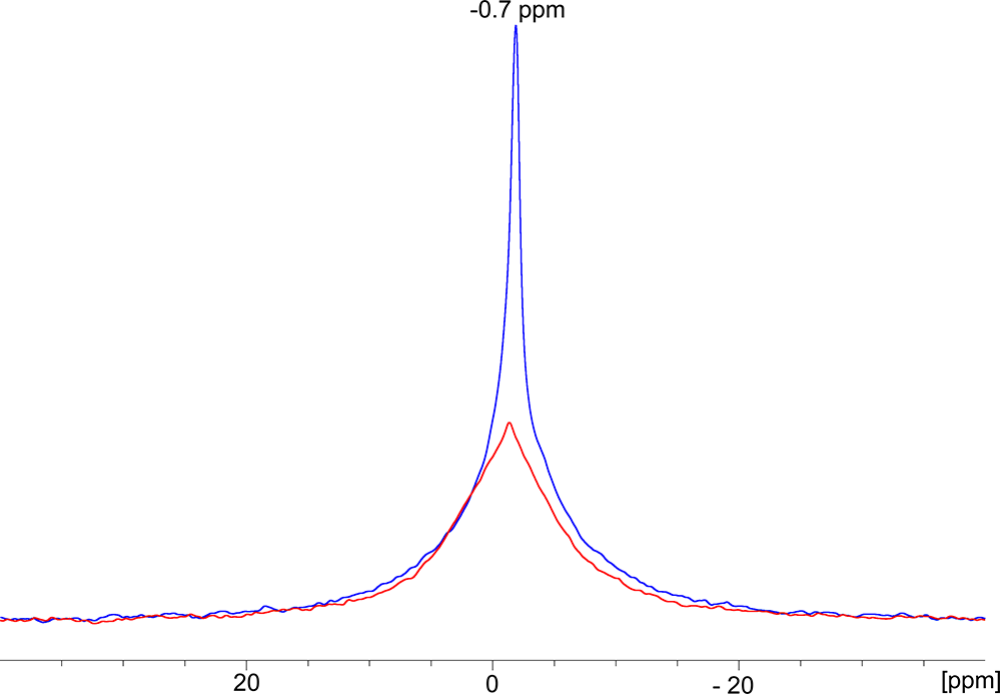
^7^Li ssNMR of c-AcH-treated Cu soaked in LP30 for 1 h (red) and 18 h (blue). The spectra were acquired with a MAS frequency of 25 kHz.

The ^7^Li NMR spectra for N-SEI on Cu flakes consist of single, relatively broad resonance in the range 10 to −10 ppm (Figure 6 and Figure S6) and correspond to various diamagnetic Li-containing species within the SEI.^45^ The ^7^Li resonance for the short soaking time (1 h) of c-AcH-Cu is significantly broader compared to that for the long soaking time sample (18 h) (Figure 6). However, for d-HCl-Cu, this trend is less significant (Figure S6). The resonances of all N-SEI samples were fitted with the combination of a broad and a narrow component, and both have approximately the same shift (Figure S7). The shift of the center of the sharp peak in Figure 6 is at −0.7 ppm. The narrowing of the resonance with soaking time could be due to a decrease in the variety of Li compounds or more likely due to the formation of more mobile Li species.^47^ The distribution of shifts seen in the 1 h spectrum is larger than the typical chemical shift range for Li, suggesting that a contribution to the broadening comes from either paramagnetic Cu^2+^ ions or bulk magnetic susceptibility effects due to Cu metal. Thus, the narrowing of the resonance with soaking time could indicate a thickening of the N-SEI on Cu over time, where the Li-containing species are further away from the Cu surface and experience reduced bulk magnetic susceptibility effects. The smaller broadening of the ^7^Li resonance on the 1 h d-HCl-Cu sample (Figure S6) could be due to faster passivation of d-HCl-Cu. Interestingly, the deconvolution of the ^7^Li resonance did not require the inclusion of a peak at −3 ppm (the typical shift of the LiFP_6_ salt^48^) to fit the spectrum.

The ^7^Li NMR spectrum for N-SEI is compared to those of the e-SEI preformed at 2, 1.4, and 0.1 V voltage holds followed by Li plating of 0.5 mAh cm^–2^ at 1.2 mA cm^–2^ in Figure S8. For the e-SEI samples, the ^7^Li resonances broaden at lower applied voltages. The ^7^Li spectra of e-SEI on Cu were fitted with 2–3 components (Figure S9), with a peak at around −1 ppm (likely corresponding to LiF) and at 0 ppm (likely Li_2_CO_3_).^45^ The third component typically had a positive ^7^Li shift (∼0.6/∼3 ppm) and can be assigned to either LiOH, Li_2_O_2_, or Li_2_O (Figure S9).^45,48^ The broadening of the ^7^Li resonance relative to the aged N-SEI resonance and the appearance of the additional resonances indicate an increase in the diversity of the Li compounds in the e-SEI.^47^

### Li Plating *In Situ* NMR

**Figure 7:**
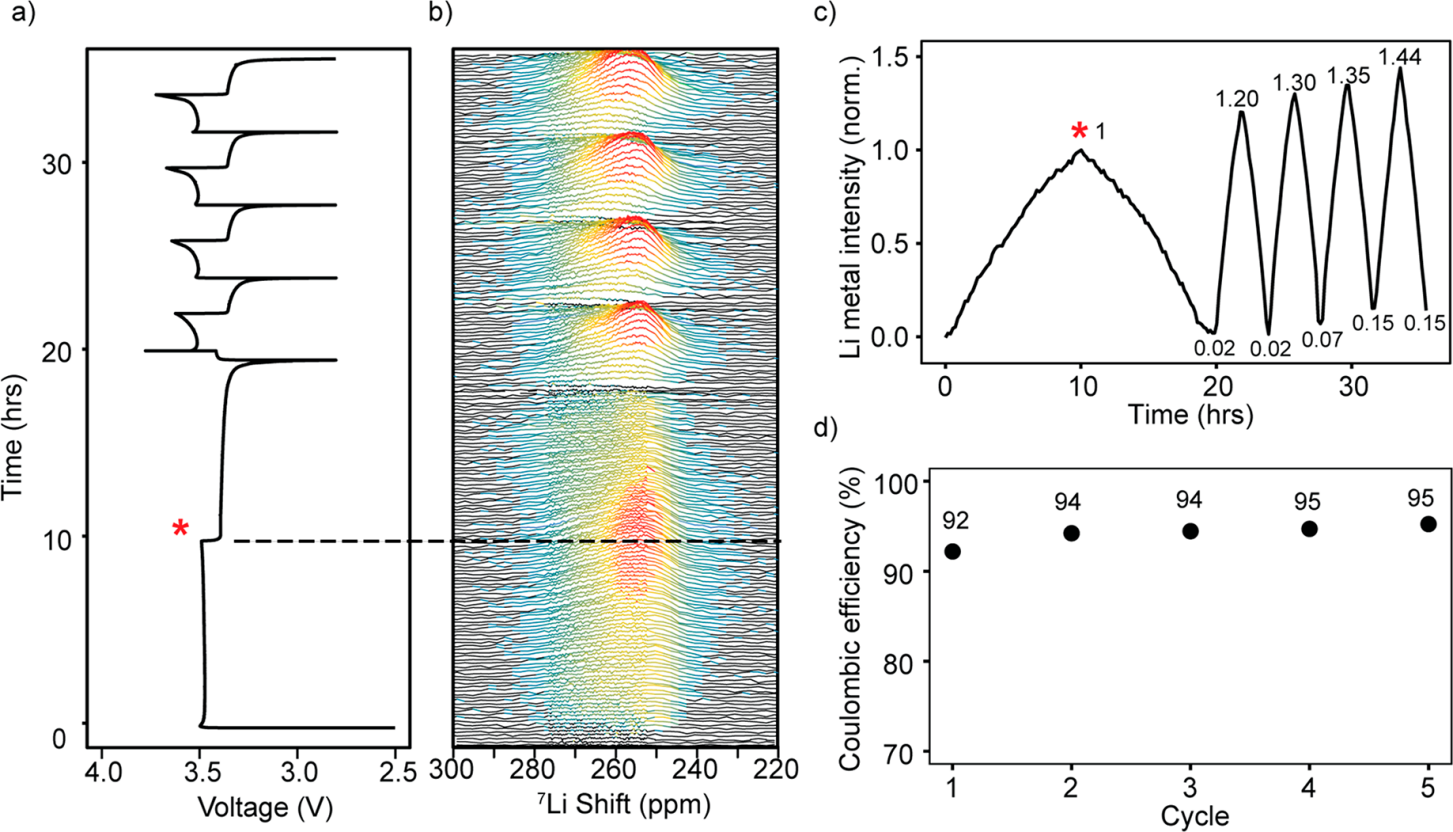
(a) Galvanostatic cycling of *in situ* Cu-LFP cells using d-HCl-Cu where a current of 0.1 mA cm^–2^ was used for the first cycle and 0.5 mA cm^–2^ for subsequent cycles; plating and stripping were performed for a constant capacity of 1 mAh cm^–2^. (b) *In situ*^7^Li NMR spectra obtained under galvanostatic cycling for d-HCl-Cu. (c) The intensity detected by NMR of the Li metal resonance, integrated over the 220–300 ppm region, the intensity being normalized to the intensity of the Li metal signal obtained on the first charge (indicated by an asterisk). (d) Coulombic efficiency for Li plating on d-HCl-Cu.

Measurements using *in situ* NMR were performed on Cu-LFP cells to study the plating and stripping of Li by using the method developed in our previous work.^24^ Before plating, the ^7^Li NMR spectrum (Figure S22a) shows the signals from the diamagnetic species close to 0 ppm (i.e., the electrolyte and the SEI) and the broad signal of the LFP. Upon plating, a new signal at ∼260 ppm emerges corresponding to the Li metal deposits, the shift to higher frequencies being a result of the Knight shift (Figure S22b).^49,50^Figure 7b shows the Li metal signal during electrodeposition on d-HCl-Cu, which grows in intensity during the plating and decreases upon stripping.^24^

Integrating the ^7^Li spectrum over the region of 220–300 ppm gives the total intensity of the Li metal signal detected by the NMR measurement (Figures 7c). The Li metal peak still remains at the end of discharge, indicative of the formation of dead Li.^24^ Upon further cycling, the intensity of the Li metal peak seen at the end of stripping grows, indicating further accumulation of the dead Li in the cell, amounting to around 15% of the initial plated Li in the fifth cycle (Figure 7c). The Coulombic efficiency (CE) of plating and stripping is 92–95% (Figure 7d). Additional experiments with either d-HCl-Cu or c-AcH-Cu gave rise to similar trends (Figures S23 and S24).

In our previous work, the capacity loss in anode-free batteries was attributed to the combined effects of SEI, dead Li formation, and galvanostatic corrosion, which all affect the degree of Li trapping on the anode.^24^ Because the LFP cathode is in large excess, and the charge passed in each plating half-cycle is constant, the changes in Li intensity reflect the three processes: Li plating (Li_plate_), Li lost to SEI formation Li_SEI_, and Li corrosion, Li_corr_, that is, the Li signal = Li_plate_ – Li_corr_ – Li_SEI_, as discussed in our previous publication. Here, the Li intensity measured by NMR is normalized to the first plating cycle; thus, an increase in plating efficiency (i.e., reduction in Li_SEI_) or in the extent of corrosion in the following cycles is expected to result in a Li intensity increase. For the first cycle, the CE, as measured by galvanostatic cycling, is 92%, growing to 94% as the current is increased and then 95% after four cycles. The dead Li measured by NMR is 2% of the initial intensity after the first cycle, whereas the Li metal signal grows by 20% between the first and second cycle. This increase in Li metal intensity cannot be explained solely by the larger loss in Li metal signal due to SEI formation in the first cycle, since the differences in the CE efficiency are not large enough; thus, galvanostatic corrosion must play an important role in the first cycle. At the end of the fifth cycle, the intensity has increased by 44% and can be explained by the accumulated capacity loss due to the SEI formation and increase in the dead Li (amounting to 15% of the initial intensity).

### SEM

**Figure 8:**
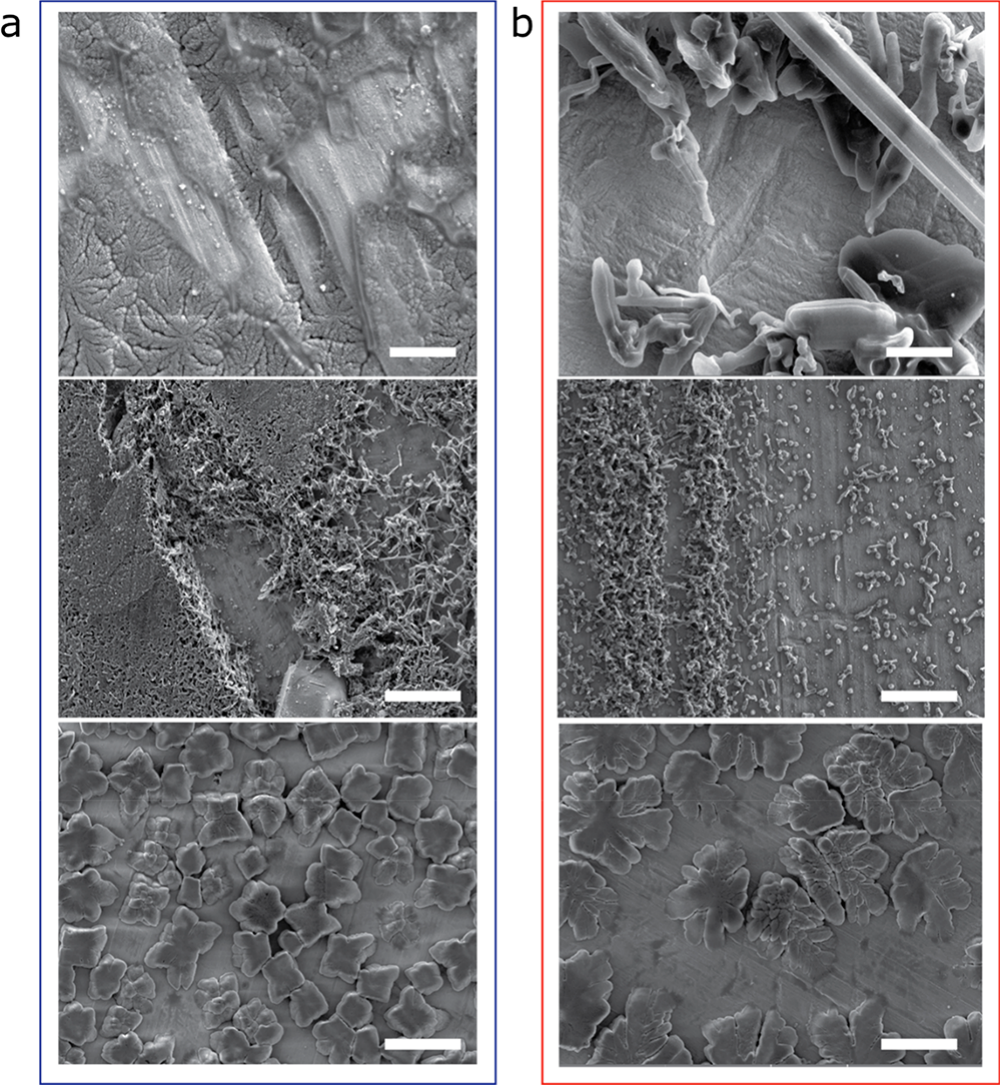
SEM of plated lithium microstructure. SEM images of lithium microstructures deposited on (a) c-AcH-Cu and (b) d-HCl-Cu disks using the procedure depicted in Figure 1a with a current density of 0.03 mA cm^–2^ (top) or 1.2 mA cm^–2^ (middle and bottom), after either 1 h rest (top and middle) or 12 h rest (bottom) at OCV, after cell assembly. The scale bars are 2 μm in the top two images and 10 μm in the middle and bottom two.

The effect of the Cu pretreatment on the resulting Li metal morphology was studied by SEM (Figure 8). Li was plated on Cu after a short rest time (<1 h) at both low and high current densities (0.03 and 1.2 mA cm^–2^, Figure 8, top and middle). Lower plating current gave rise to a smoother and denser Li morphology (Figure 8, top). The coverage of the Cu substrate by Li is better on c-AcH-Cu; thus, Li plating on c-AcH-Cu is more homogeneous compared to d-HCl-Cu (Figures 8a and 8b, respectively). When Li is deposited on c-AcH-Cu, Li plating takes place both in the trenches and on the surface of the Cu foil (Figure 8a). The ability to produce electroplated coatings of uniform thickness on samples with a complex geometry is termed “high throwing power”^51^ and is typical for cases where the surface film is significantly more resistive compared to the electrolyte. In an SEI-based system this condition is fulfilled for electrodes passivated with a homogeneous SEI. When the N-SEI formation time prior to plating is extended to 12 h and plating is then performed at 1.2 mA cm^–2^, thicker Li deposits are formed (Figure 8, bottom).

### XPS on Li-Plated Cu

**Figure 9:**
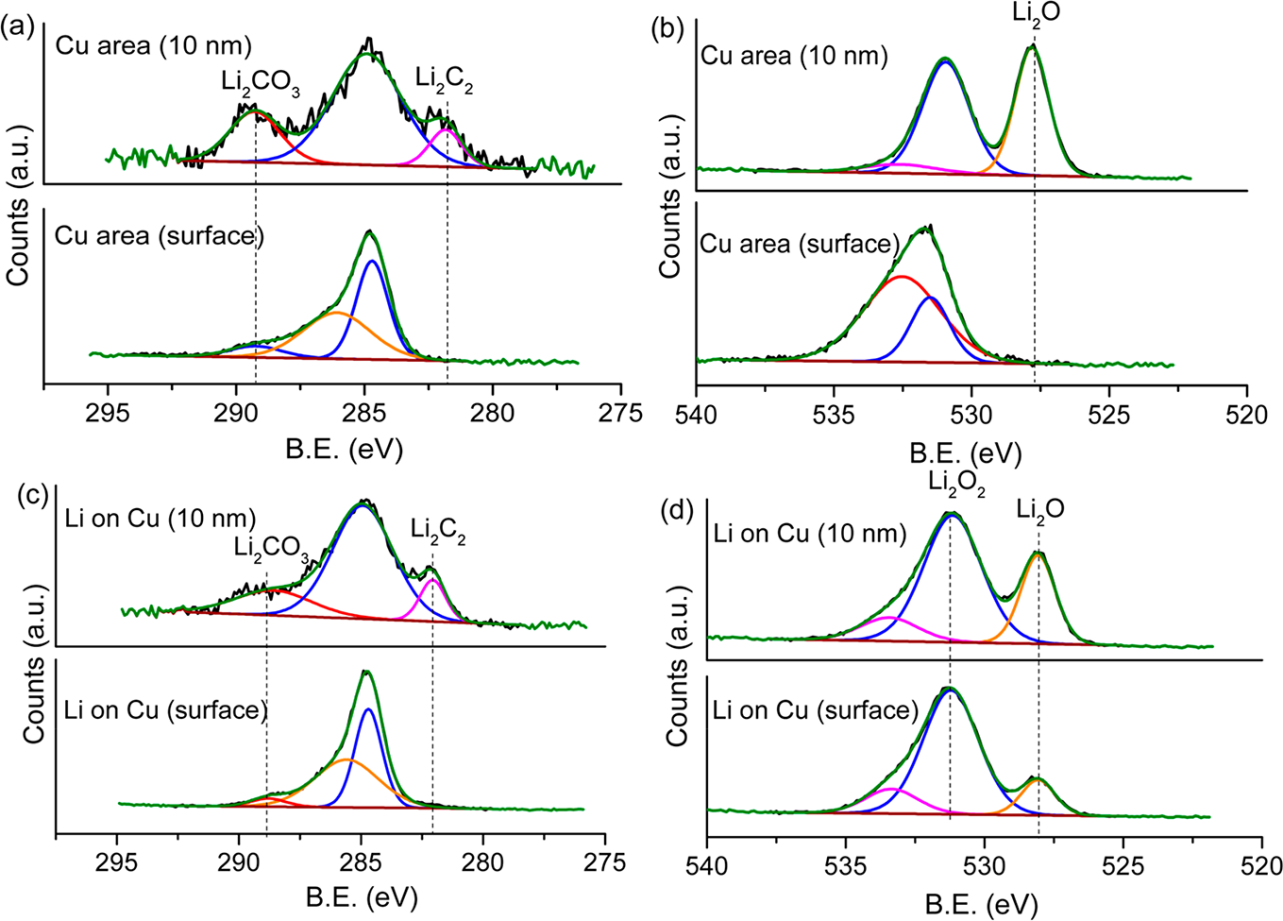
XPS of SEI formed at a constant current of 0.03 mA/cm^2^ (using the procedure depicted in Figure 1a) on exposed Cu (a) C 1s, (b) O 1s, and Li-plated Cu (c) C 1s and (d) O 1s on the d-HCl-Cu film.

To understand which compounds in the SEI encourage Li plating, the spatial distribution of the SEI was next investigated by using XPS on the samples prepared following Li plating on d-HCl-Cu foils. The measurements show how both the SEI on the exposed Cu surface and the plated lithium consist of similar carbon composites, with similar C 1s spectra (Figures 9a and 9c). The C 1s spectrum is dominated by C–C/C–H and −C–O and −O–C=O species on the surface, while on 10 nm sputtering additional peaks appear at 289.3 and 282.2 eV, mainly due to the presence of Li_2_CO_3_ and Li_2_C_2_, respectively.^18^

The O 1s spectrum of the exposed Cu surface changes significantly upon ∼10 nm sputtering, revealing a new peak at 528 eV (Figure 9b), attributed to Li_2_O.^41,52^ The Li_2_O peak at 528 eV was also observed on the O 1s spectrum of lithium-plated areas, both on the surface and after ∼10 nm sputtering (Figures 9b and 9d). The presence of Li_2_O was further confirmed by the Li 1s peak at 53–54 eV (Figure S21a).

The O 1s spectrum of SEI on the exposed Cu area (Figure 9b) consists of two broad peaks at 531.5 and 532.5 eV, assigned to OH^–^ and Li_2_CO_3_ at the surface. After sputtering 10 nm, the OH^–^ peaks dominate (at around 531.0 eV).^41,53−56^ The increased concentration of OH^–^ was observed on the exposed Cu surface by ToF-SIMS (Figures S29a, S30, and S31).^6,41^

The O 1s peak at 531.2 eV, observed exclusively on the Li-plated areas, corresponds either to LiOH or to Li_2_O_2_ (Figure 9d), the O 1s and Li 1s characteristic binding energies for both Li_2_O_2_ and LiOH being very close.^41,52^ However, since Li_2_O_2_ is known to be more stable in the XPS beam,^41^ and since this signal persisted during sputtering (Figure 9d), this peak was tentatively assigned to Li_2_O_2._

### ToF-SIMS

**Figure 10:**
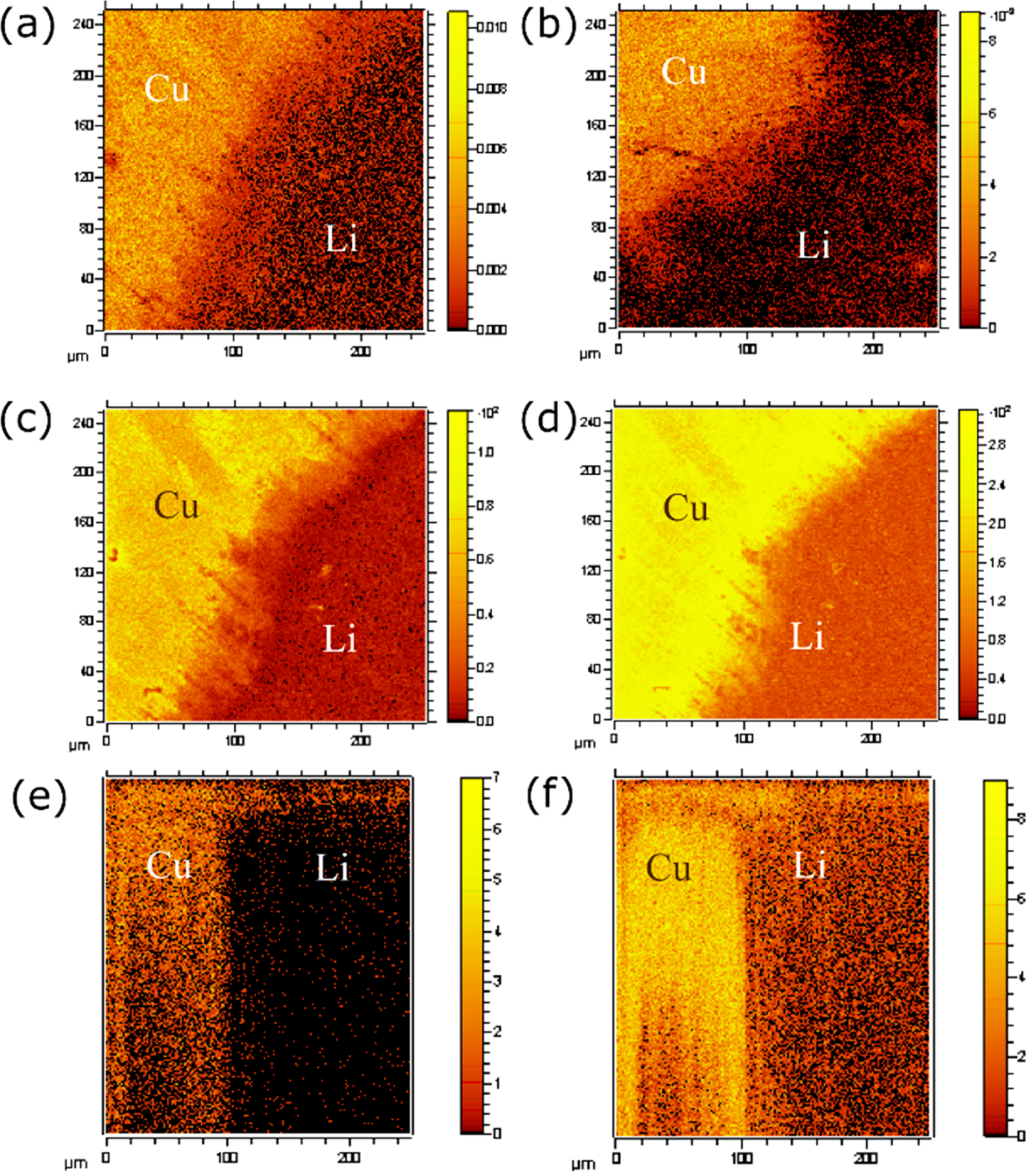
ToF-SIMS maps of Li plating on Cu films. CuO (^65^CuO^–^) map of SEI on Li-plated Cu for (a) d-HCl-Cu and (b) c-AcH-Cu. Maps of (c) LiF_2_^–^ and (d) OH^–^ and (e) 3D reconstruction (*XZ*-plane) of LiF_2_^–^ and (f) OH^–^ of eSEI_0.1V_ on d-HCl-Cu. Images a–d are point-to-point normalized to total counts. SEI on plated lithium and exposed copper areas is labeled as Li and Cu, respectively.

ToF-SIMS was used to achieve a deeper understanding of the effect of voltage-specific SEI compositions on the Li plating. eSEIs were formed at a constant voltage in the range 2–0.1 V, and then 0.1 mAh of Li was plated on the Cu at 1.2 mA cm^–2^ (SEI formed at constant voltage *X*V is denoted eSEI_*X*V_, for example, eSEI_0.1V_ for SEI formed at 0.1 V). The voltage profile for the preparation of the samples for ToF-SIMS is depicted in Figure 1b. CuO, LiF, and OH^–^ (detected as ^65^CuO^−^, LiF_2_^–^, and OH^–^, respectively) were mapped on a 250 × 250 μm^2^ area of Li plated samples (Figure 10 and Figures S28–S33). The mapping was done on an area on the sample that visibly contained both areas of plated Li and exposed Cu surface.

The CuO maps on Li-plated and exposed Cu areas of the films are depicted in Figures 10a and 10b. Interestingly, significant CuO^−^ signal was observed on the areas containing lithium metal, which indicates that Cu_*x*_O is present in the SEI on the Li metal. Furthermore, the intensity of the Cu oxides on the Li metal is noticeably higher on the Li plated on d-HCl-Cu compared to the Li on c-AcH-Cu. To determine whether the Cu oxide signal originated in the Cu current collector or the Cu oxide in the SEI, 3D reconstructions of the CuO maps were performed (see Supporting Information for 3D reconstruction at the *XZ*-plane, Figure S28). Because the CuO^−^ signal was observed throughout the depth profile of the SEI, it appears that Cu oxides are intermixed with the SEI and are found even in its outer layers.

The hydroxide group (OH^–^) was found to give rise to high-contrast maps and vastly different depth profiles when comparing plated and nonplated areas (Figure 10d,f, Figures S29a, S30, and S31). The OH^–^ map of e-SEI_0.1V_ on the Li-plated d-HCl-Cu sample (Figure 10d) is depicted alongside its 3D reconstruction (*XZ*-plane) (Figure 10f). A significantly higher intensity of the OH^–^ signal was observed for the SEI on the exposed Cu areas. Similar maps were shown for both e-SEI_2V_ and e-SEI_0.1V_ on c-AcH-Cu and d-HCl-Cu samples (Figures S29a, S30, and S31). The top surface of both areas is covered by a thin layer of OH^–^ (Figure 10f, Figures S30b and 31b). This layer is attributed to OH^–^ which precipitated after the Li plating. Underneath the top layer there is a decrease in the intensity of the signal, presumably due to precipitation of SEI compounds that do not have an OH^–^ group. Deeper layers of the *YX*-plane reveal a significantly higher intensity of the OH^–^ signal on the nonplated surface for both d-HCl-Cu and c-AcH-Cu samples.

The map of LiF (detected as Li_2_F_3_^–^ or LiF_2_^–^) signal in the e-SEI_0.1V_ on d-HCl-Cu (Figures 10c and 10e) shows that, similar to the OH^–^ signal, the intensity of the LiF signal is consistently higher in areas with no plated Li. This suggests that there is preferential plating of Li in areas where the concentrations of LiF and OH^–^ are lower—a proposal that is discussed in more detail below. A similar trend was observed for the LiF map of c-AcH-Cu samples (Figures S29b, S32b, and S33b).

## Discussion

The spontaneous formation of the N-SEI on the surface of Cu and Cu oxides, regardless of the surface treatment, was confirmed by ^19^F and ^7^Li ssNMR, EIS, XPS, and ToF-SIMS. The N-SEI was shown to be mainly composed of LiF and LiPF_6_ decomposition products in addition to copper oxides. The formation of LiF on Cu at the OCV is assigned to the chemical decomposition of LiPF_6_ due to its reaction with trace amounts of water (reactions 2 and 3).^37,46,57−59^
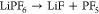
2
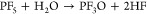
3Analysis of the ssNMR spectra revealed the existence of ^19^F NMR resonances at around −142 and −225 ppm. While the resonance at −225 ppm is solely seen for the N-SEI, the resonance at −142 ppm is observed for both N-SEI and e-SEI_2V_ (Figure 5 and Figures S3–S5). Recently, Lebens-Higgins et al.^61^ assigned the ^19^F NMR resonances around −144 ppm to F^–^ ions bound to the surface of transition metal cathodes. Clément et al.^60^ also reported a similar ^19^F shift at −144 ppm, which was ascribed to lithiated fluoride on the electrode surface.^61^ Here we tentatively assign both resonances (−144 and −225 ppm) to free fluoride ions in various environments and proximities to the Cu oxide surface. Different chemical environments of the fluoride could be a result of the formation of various combinations of ion pairs and triplets, solvation of the fluoride ion, and formation of hydrogen bonds to the OH^–^ group on the Cu surface.^62,63^

The variation in the OCV of the Li–Cu half-cells is attributed to the interplay between the surface bonded Cu_*x*_O, CuF_2_, and the N-SEI. The ^19^F NMR resonance at −142 ppm was not observed for e-SEI formed at either 1.4 or 0.1 V, the free fluoride ions presumably reacting further to form LiF.

The XPS analysis of the N-SEI indicates that it is composed of Cu oxides, LiF, and LiPF_6_ decomposition products (Li_*x*_PF_*y*_, LiPO_*x*_F_*y*_), while the e-SEI formed at 2 and 1.4 V contains additional PEO-like compounds (Figures S16–S19). The sequential reactions 4 and 5 represent a possible reaction mechanism for the formation of LiF, Li_2_O, and OH^–^.^22,42,64,65^
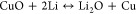
4
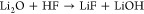
5Upon comparison of the LiF:LiPF_6_ ratio in the F 1s spectra, it seems that LiF is the main species in both the N-SEI and e-SEI_2V_, while for the e-SEI_1.4 V_, LiPF_6_ derived species (Li_*x*_PF_*y*_, LiPO_*x*_F_*y*_) are more dominant on the surface, with the LiF signal only observed after sputtering (Figures S16 and S17, Table S2). This could be the result of the formation of LiF both chemically and electrochemically mainly during rest or at the first stages of SEI formation. This assumption is further supported with the LiF ToF-SIMS depth profile, in which the intensity of the LiF signal is significantly higher for e-SEI formed at either 2.8 or 2 V (Figure S27a).

A thicker SEI forms at 2–1.4 V and is mainly composed of LiF, Li_2_O and CuO. The Li_2_O signal, observed in both Li-plated and exposed Cu areas, is an indication for reduction of the Cu oxides prior to or during Li plating (Figures 9b and 9d).^33,41^ The masking of the Cu 2p and O 1s signals of Cu oxides and the negative shift of the Cu 2p peaks were assigned to the lithiation of the Cu oxides (Figures S15 and S20), which could be a possible reason for the thickening of the SEI at 1.4 V.^31^ In addition, LiF could be a product of the Cu oxide reduction at 2–1.4 V (reactions 4 and 5).^22^

Li_2_O_2_ was observed on Li metal microstructures plated on Cu (Figure 9d). Li_2_O_2_ was previously proposed as an intermediate in Cu oxide reduction reactions.^26,27,39^ However, the Li_2_O_2_ could also be a product of the reaction of high-surface-area Li microstructures with trace amounts of oxygen found in the glovebox or present during sample transfer. The latter is more likely since there is no strong driving force to form peroxides from oxides at these voltages; moreover, the Li_2_O_2_ signals were measured only on the Li metal microstructure interface.

LiOH was observed in the O 1s XPS spectrum (Figure 9b) on the exposed Cu areas.^41,52−54^ Because the OH^–^ signal was not measured on the Li-plated areas, it was concluded that OH^–^ on the surface of Cu discourages Li plating, as was further confirmed by ToF-SIMS.

Li carbide (Li_2_C_2_) was found by XPS (Figures 9a and 9c) in the inner layer of the SEI. Li_2_C_2_ was observed previously on Li-plated Cu.^3^ However, contrary to the observations of Eshetu et al.,^18^ here it was observed on both the Li-plated and exposed Cu areas. Li_2_C_2_ was observed on Li-plated samples by ToF-SIMS as well; however, because of the presence of several substances with similar masses in the SEI, the signal of Li_2_C_2_ cannot be normalized to the total ion concentration. These findings agree with the findings of Schmitz et al.,^3^ and it is likely formed by the reduction of Li_2_CO_3_^66^ or organic SEI components. Further study is required to understand the role of nonstoichiometric Li_2_C_2_ formation during Li plating.

Cu_x_O was found by using ToF-SIMS in the SEI on both Li-plated and exposed Cu areas (Figure 10a and 10b). The intensity of Cu oxides on the surface of Li plated on d-HCl-Cu is noticeably higher. These findings suggest that Cu oxides are transported from the N-SEI to the SEI formed electrochemically. Likely, this results from a combination of Li plating underneath some of the initial electronically insulating N-SEI and copper oxide dissolution in acids created by reactions of water with PF_6_^–^ and then reprecipitation either reductively as the metal or in the SEI salts as the cations.^67^ However, since the intensity of CuO^−^ is similar on the Li plated and exposed Cu areas, Cu oxides do not seem to be the cause of preferential Li plating.

Aging of the N-SEI gives rise to an increase in the heterogeneity of the SEI, as shown by using EIS (Figure 2 and Figure S2). The increased heterogeneity results in the formation of “hot spots” in the SEI, which results in preferential plating on these spots and uneven Li plating (Figure 8, bottom).

The intensity of the metal Li NMR signal was measured by using *in situ* NMR during plating and stripping in LFP-Cu cells. The Li metal peak remains at the end of Li stripping, indicative of the formation of dead Li, as previously reported.^24,68^ Furthermore, a substantial increase in Li metal signal intensity is also seen at the end of plating. There are two possible explanations for this increase between cycles: (i) The formation of dead Li, as a result of inhomogeneous stripping, increases the amount of Li metal in the cell. (ii) A significant amount of the charge passed in the first cycles is used to form the SEI; thus, in the subsequent cycles the total amount of Li metal in the cell increases. Because the amount of accumulated dead Li and the SEI formation cannot fully explain the growing amount of Li at the end of each charge, the additional efficiency loss was assigned to galvanic corrosion of Li on the Cu surface. Furthermore, quasi-reversible parasitic reactions, which were observed in the CV measurements (Figure S25), are also likely to contribute to the Li capacity loss in anode-free Li batteries.

A ToF-SIMS study of the SEI composition revealed preferential Li plating on OH^–^ and LiF deficient areas. The signals of OH^–^ and LiF are consistently higher from the SEI on nonplated Cu compared to the ones from the SEI on Li metal for both Cu treatments. The OH^–^ and LiF signals from N-SEI on c-AcH-Cu are lower and more homogeneous throughout the depth profile (Figure S27). The smoother Li morphology on c-AcH-Cu is attributed to more homogeneous distribution of OH^–^ and LiF on N-SEI.

## Conclusions

A native, inorganic SEI-like layer, composed of LiF, LiPF_6_ decomposition products, Cu oxides, and fluorides, spontaneously forms on Cu current collectors upon their immersion in a LiPF_6_-based electrolyte. LiF in the native SEI is formed by multistep reaction of LiPF_6_ with trace amounts of water, and exists both in solid form and as free Li^+^ and F^–^ ions. The passivating properties of the N-SEI are influenced by the Cu oxide layer’s homogeneity and chemical composition. The OCV of the cells is determined by the copper interface composition, homogeneity, and formation time. The electrochemically formed SEI on Cu is composed of N-SEI as the primary layer, Cu oxide reduction and lithiation products (Li_*x*_CuO, Li_2_O, and LiOH), and solvent reduction products.

Copper oxides are mixed in the SEI during its formation and transferred to the anode–electrolyte interface. The reduction of Cu oxides at the interface determines both the composition of the SEI prior to Li plating and SEI evolution during battery cycling. The presence of transition metal oxides in the SEI might encourage the thickening of the SEI during cycling and, as a result, cause rapid degradation. *In situ* NMR was used to study Li plating efficiency on Cu, revealing parasitic reactions which contribute to capacity loss, in addition to SEI and dead Li formation.

The preferential plating seen with both XPS and ToF-SIMS is governed by the distribution of low ionic conducting compounds (LiF, LiOH, and Li_2_O) rather than electronic conducting compounds (Cu, CuO, and Li_2_C_2_). Thus, we suggest that the cause of preferential plating is the significant variance in the ionic conductivity of SEI components giving rise to heterogeneous current density distribution on the Cu surface, which results in heterogeneous Li coverage and morphology.

Li plating on Cu is affected by the interplay between the SEI components and SEI homogeneity. A homogeneous SEI composition will give rise to a homogeneous current density and smoother Li morphology. Because an OH^–^-rich Cu surface encourages SEI homogeneity, it is preferred in spite of its higher impedance. Thus, the choice of the current collector and its treatment is critical for the realization of practical “anode-free” batteries.

## Supporting Information Available

Details on the fitting of the EIS spectra; additional ssNMR spectra for the different SEIs, formed at different experimental conditions, additional XPS spectra, additional *in situ* NMR data on the Li plating and the electrochemical cycling data, CV plots of further Li plating and stripping cycles, additional ToF-SIMS depth profiles and maps, table of the peak energies and molecular weights used in the assignment of the XPS and ToF-SIMS spectra, respectively (PDF)
