# Supplementary material for: Toward an Understanding of SEI Formation and Lithium Plating on Copper in Anode-Free Batteries
Source: J Phys Chem C Nanomater Interfaces. 2021 Jul 27;125(30):16719–32. doi: 10.1021/acs.jpcc.1c03877 (PMC8392351; doi:10.1021/acs.jpcc.1c03877)
Supplement: Supplementary file 1 — jp1c03877_si_001.pdf [file jp1c03877_si_001.pdf]

Supplementary Information
Towards an understanding of SEI formation and lithium plating on copper in anode-free batteries

Svetlana Menkin[1], Christopher A. O'Keefe[1], Anna B. Gunnarsdóttir[1], Sunita Dey[1], Federico M. Pesci[2], Zonghao Shen[2], Ainara Aguadero[2], Clare P. Grey[1*]

1. Department of Chemistry, University of Cambridge, Lensfield Road, Cambridge, CB2 1EW
2. Department of Materials, Imperial College London, Royal School of Mines, SW7 2AZ

Supplementary information

EIS study of N-SEI **1**

^{19}F and 7Li SSNMR **4**

XPS **11**

***In situ* NMR** **19**

TOF-SIMS **21**

EIS study of N-SEI

The EIS Nyquist plot of d-HCl-Cu symmetrical cells is typical for a linear restricted diffusion towards a blocking electrode (Figure 2a in the manuscript).

The plots of c-AcH-Cu symmetric cell were fitted with a Randomize + Simplex fitting method with 200,000 iterations for each step. The Nyquist plot measured after one-hour rest time, was fitted with Voigt-type equivalent circuit a R1+Q1/(R2+Wd1)+Q4/R4 (Figure S1, Table S1). R1 was assigned to the impedance of the bulk electrolyte. R2, R4 and Q1, Q4 were assigned to the impedance and capacitance of different phases in the N-SEI. The confined finite length diffusion component (Wd), typically assigned to mass transport through a layer with a finite length, was assigned to the mass transfer in the N-SEI (Figure S1, Table S1).

An equivalent circuit composed of multiple RC units was previously assigned to SEI.[1] The maximum of the high frequency semi-circle is at approximately 165 kHz and its capacitance is of the order of 10^{-9} F (Figures 2b and S1, Table S1). The semi-circles at frequencies above 100 kHz, are typically assigned to the compact surface films adjacent to a metal surface.[1–3] The

relatively low capacitance of the semi-circle (around 10^{-9} F) also could be due to the significant roughness of the Cu substrate. [4]

After 5 days' rest time, the fit of the impedance spectrum Nyquist plot for c-AcH-Cu required an additional RC component, assigned to the SEI. The diameter of the high-frequency semi-circle decreases with time, while the total impedance has not changed significantly. The slope of the Nyquist plot of d-HCl-Cu decrease with time. This indicates a relative increase in the time constants of the diffusional as compared to the faradaic processes.[5] We attribute these trends to an increase in the heterogeneity of the SEI after prolong rest time. [6,1]

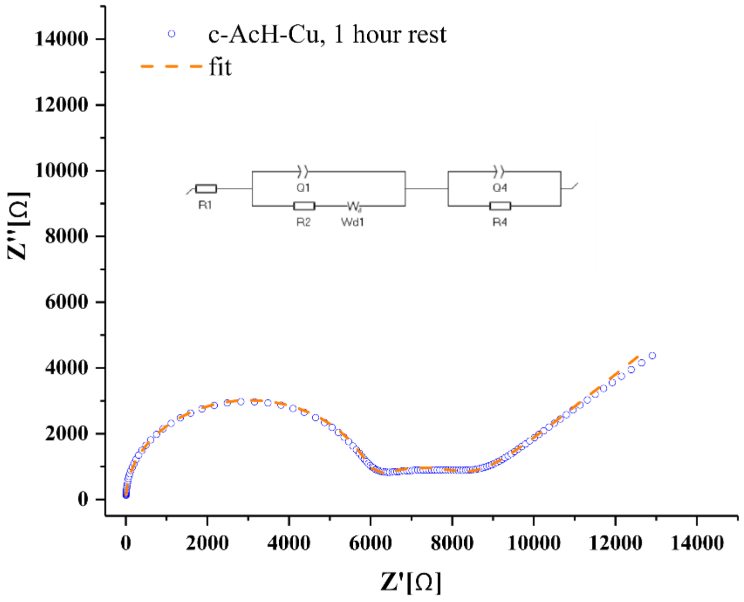

Figure S1. PEIS in the 1 MHz – 1 Hz frequency range, with an amplitude of 10 mV, Nyquist plot in symmetric cells of N-SEI on c-AcH-Cu after 1-hour rest (blue) and the equivalent circuit fit (orange, see circuit insert).

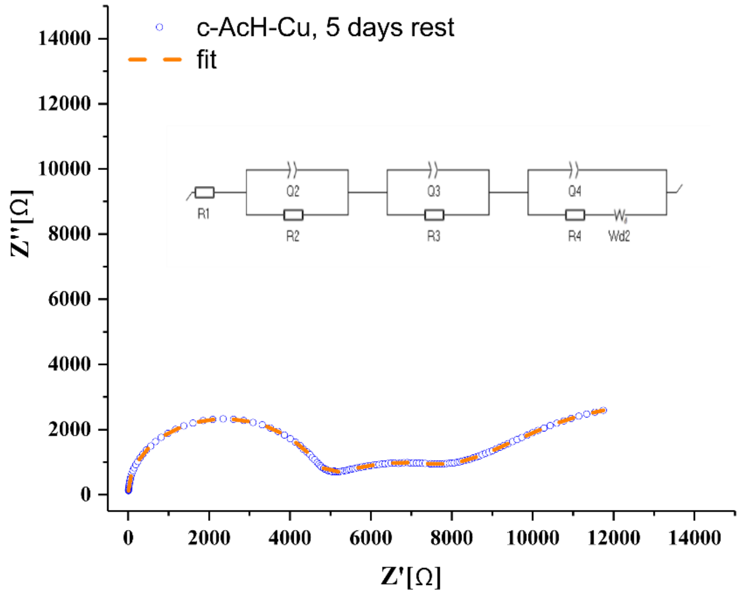

Figure S2. PEIS in the 1 MHz – 1 Hz frequency range, with an amplitude of 10 mV, Nyquist plot in symmetric cells of N-SEI on c-AcH-Cu after 5-day rest (blue) and the equivalent circuit fit (orange, see circuit insert).

Table S1. The resulting fitting parameters for the c-AcH-Cu symmetric cells shown in Figure S1 and Figure S2.

Parameter	Value for t_{rest}=1 hour	Value for t_{rest}=5 days
R2 [Ω]	2488	4571
R3 [Ω]	5897	955
R4 [Ω]	NA	3034
Q2, a2 [$Fs^{(a-1)}$]	0.4e-6, 0.75	0.2e-9, 1
Q3,a3 [$Fs^{(a-1)}$]	0.2e-9, 1	0.3e-6, 0.8
Q4,a4 [$Fs^{(a-1)}$]	NA	2.8e-6, 0.65
Rd2 [Ω] (Wd)	5897	5648
Fit $\chi/\vert z \vert$	0.04	0.03

^{19}F and 7Li SSNMR

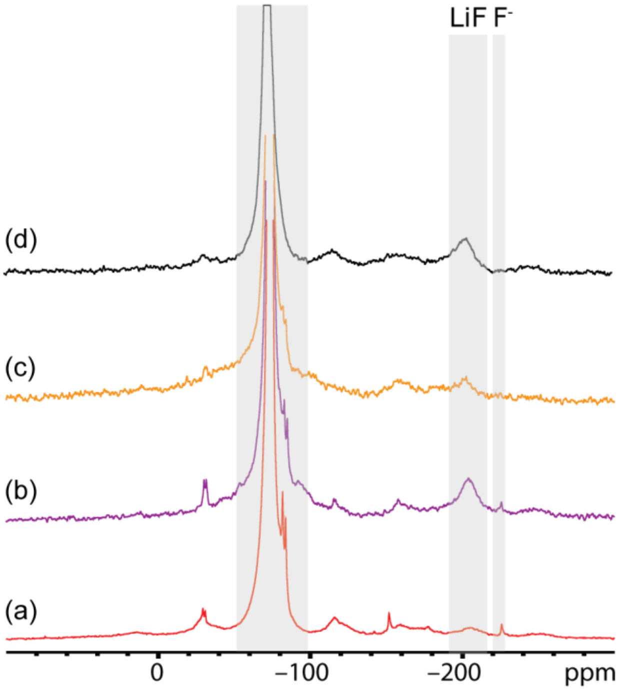

Figure S3. ^{19}F SSNMR spectra of the N-SEI on (a) d-HCl treated copper, (b) CuO, (c) Cu_2O and (d) lithium metal soaked in LP30 for over 30 hours.

The effect of soaking time was studied by comparing the ^{19}F NMR spectra of samples of d-HCl-Cu soaked for 1 hour and 96 hours and of c-AcH-Cu soaked for 1 hour and 18 hours (Figure S4). Both sets of spectra consist of resonances corresponding to $LiPF_6$ and its decomposition products and a broad LiF resonance.[7] In addition, sharp resonances around -225 ppm were observed in all spectra except c-AcH-Cu soaked for 1 hour.

In all the ^{19}F NMR spectra (Figures 5, S3-5, S10-11), a group of poorly resolved resonances in the range of -130 ppm to -180 ppm appears in varying intensities (detailed in Figure S5). The -156 ppm and -153 ppm resonances are attributed to HF in several publications.[8] However, it is not fully understood how HF can co-exist with lithium metal. The resonance around -180 ppm could be attributed to fluoride ions coordinated to hydroxide[9] or oxide species (Figure S5).[10] In addition, several of the spinning side bands for the $LiPF_6$ and LiF appear in this range, adding to the complexity of the spectrum.

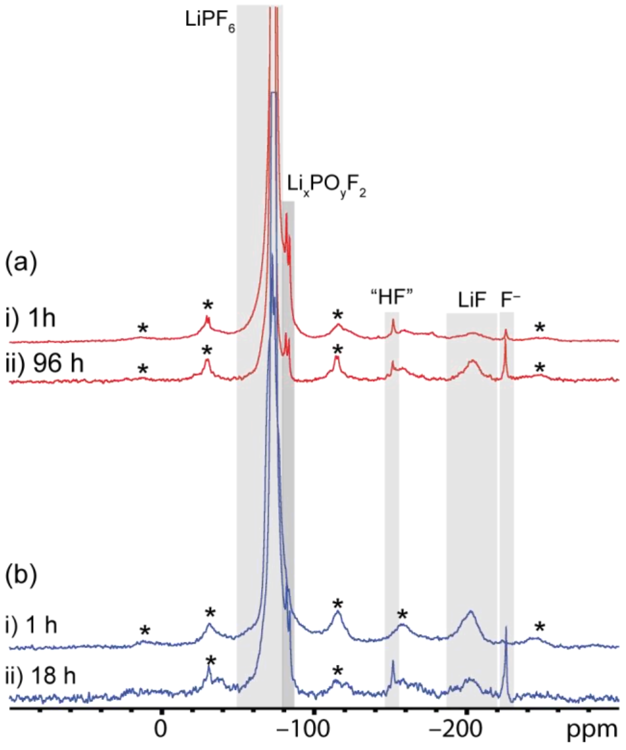

Figure S4. ^{19}F ssNMR of treated Cu flakes soaked in LP30 electrolyte. (a) d-HCl-Cu soaked for (i) 1 hour and (ii) 96 hours, (b) d-AcH-Cu soaked for (i) 1 hour and (ii) 18 hours. The spectra were acquired a MAS frequency of 25 kHz. The spinning side bands are marked with asterisks.

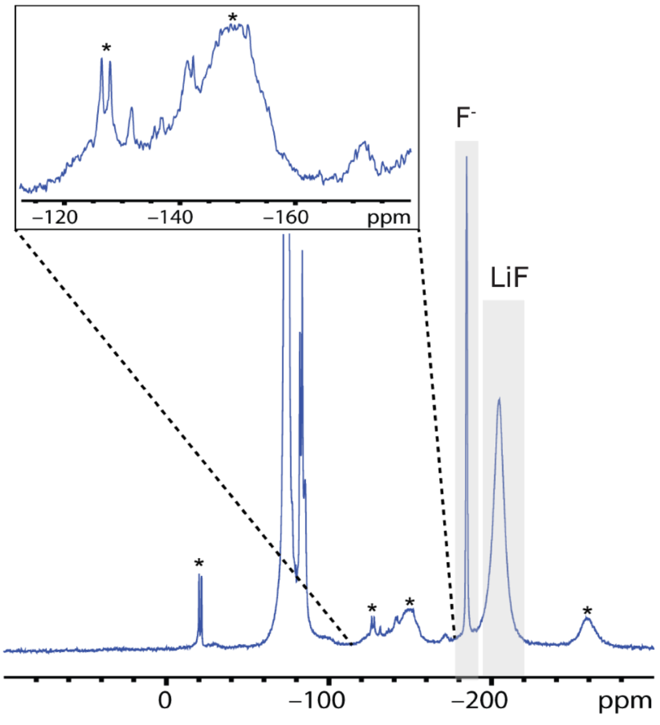

Figure S5. ^{19}F ssNMR of the N-SEI on d-AcH treated Cu soaked in LP30 for 24 hours. The spinning side bands are marked with asterisks. The spectra were acquired with a MAS frequency of 25 kHz.

The effect of N-SEI formation time is demonstrated for the N-SEI on d-HCl-Cu formed for 1 hour which gives rise to broader ^{7}Li resonance compared to that of the N-SEI formed for 96 hours (Figure S6).

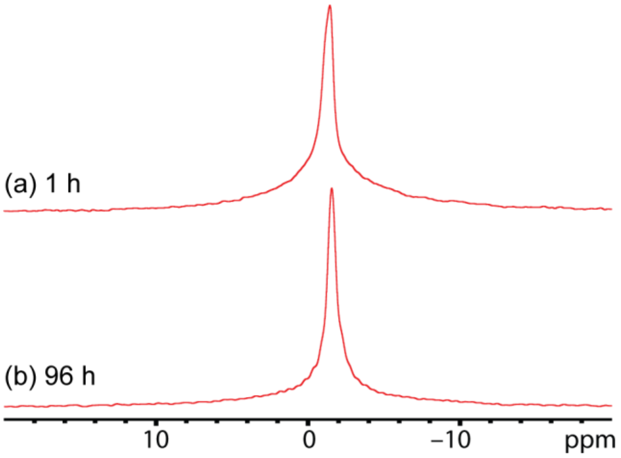

Figure S6. ^{7}Li ssNMR spectra of the N-SEI on d-HCl-Cu soaked in LP30 for (a) 1 hour and (b) 96 hours.

The resonances on all N-SEI were fitted with the combination of a broad and a narrow component, both having approximately the same isotropic chemical shift (Figure S7). The ^{7}Li NMR spectra for N-SEI on c-AcH-Cu powder is compared to those of the e-SEI pre-formed at 2 V, 1.4 V and 0.1 V voltage holds followed by lithium plating of 0.5 mAh cm^{-2} at 1.2 mA cm^{-2} on c-AcH-Cu and d-HCl-Cu (Figure S8 (a) and (b), respectively). The e-SEI_{2v} gives rise to narrower ^{7}Li resonance compared to N-SEI for both samples. The ^{7}Li spectra of e-SEI on Cu were fitted with 2-3 components, mainly LiF (~ -1 ppm) and Li_2CO_3 (~ 0 ppm). Additional components with positive ^{7}Li shifts (~ 3.0, ~3.8 ppm) were assigned to Li_2O (Figure S9). [11,12]

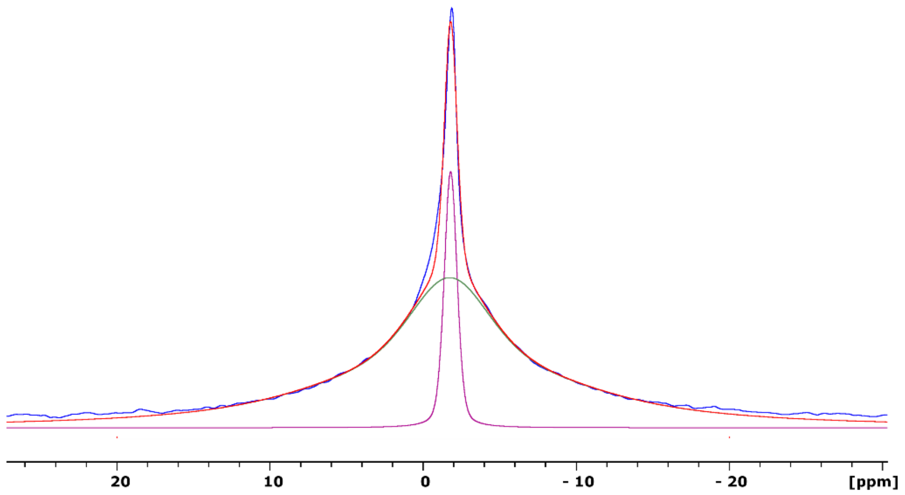

Figure S7. (a) Deconvoluted ^{7}Li ssNMR spectra of the c-AcH-Cu (flakes) soaked in LP30 for 18 hours (also shown in Figure 6). The fit consists of two components (a broad component at -0.5 ppm and a narrow component at -0.7 ppm).

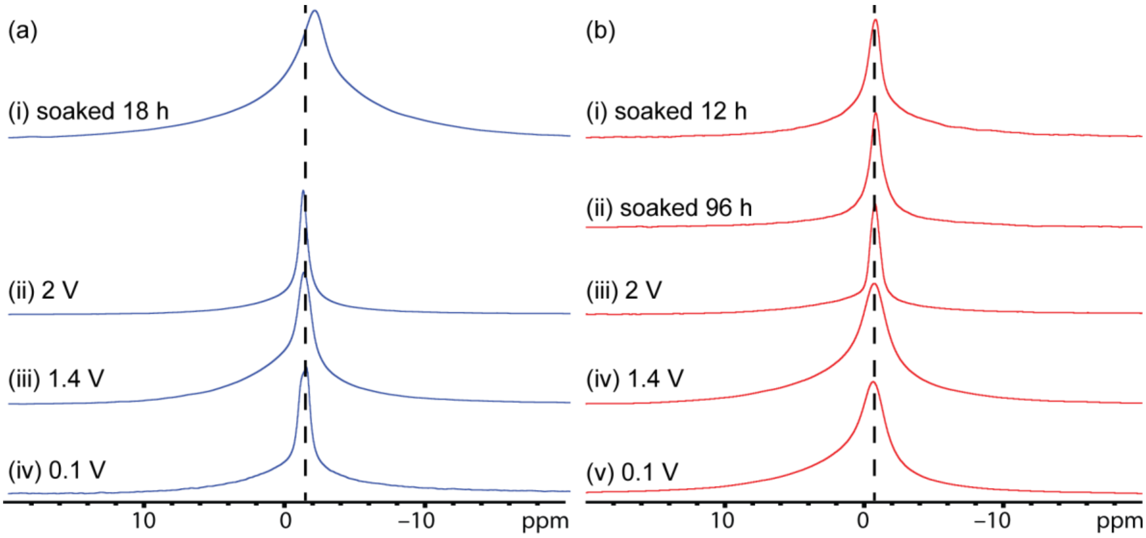

Figure S8. (a) ^{7}Li ssNMR of (i)c-AcH-Cu (flakes) soaked in LP30 for 18 hours, (ii) e-SEI$_{2V}$, (iii) e-SEI$_{1.4V}$ and (iv) e-SEI$_{0.1V}$ on lithium plated c-AcH-Cu (foil). (b) ^{7}Li ssNMR of d-HCl-Cu (flakes) soaked in LP30 for (i) 12 hours and (ii) 96 hours, (iii) e-SEI$_{2V}$, (iv) e-SEI$_{1.4V}$ and

(v) e-$SEI_{0.1V}$ on lithium plated d-HCl-Cu (foil). The spectra were acquired with a MAS frequency of 25 kHz.

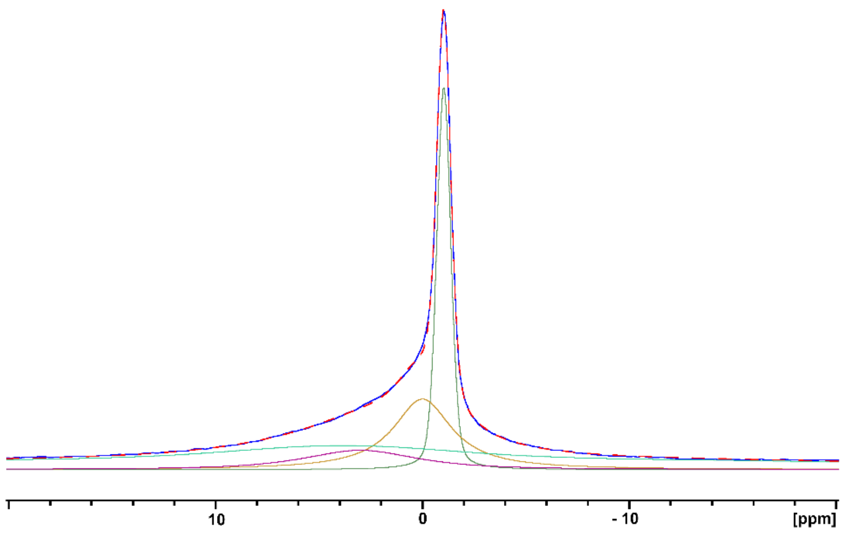

Figure S9. (a) Deconvoluted ^{7}Li ssNMR spectra of the e-SEI_{2V} (formed at 2 V for 45 hours) on lithium plated d-HCl-Cu (foil). The fit consists of four components (three broad components at 0, 3.0 and 3.8 ppm and a narrow component at -1 ppm).

The ^{19}F NMR spectra of the scraped Li metal plated on to the c-AcH-Cu and d-HCl-Cu electrodes (with voltage hold of 2V for 10 and 24 hours on c-AcH-Cu and 12 and 70 hours on d-HCl-Cu) are depicted in Figure S10. In addition to the peaks corresponding to $LiPF_6$ and LiF, sharp resonances at -96 ppm (doublet) were observed for c-AcH-Cu sample soaked for 10 hours and sharp resonances at -142 ppm and -156 ppm were observed for the e-SEI_{2V} formed for 24 hours. In contrast, the LiF peak at -204 ppm and the -142 ppm resonances were absent from d-HCl-Cu spectra. For all e-SEI_{2V} samples, the resonance at -225 ppm was not observed (Figure S9), suggesting that this resonance is typical to N-SEI. The ^{19}F NMR spectrum of e-$SEI_{1.4V}$ on both d-HCl-Cu and c-AcH-Cu (Figure S11) consists of mainly $LiPF_6$ and LiF resonances.

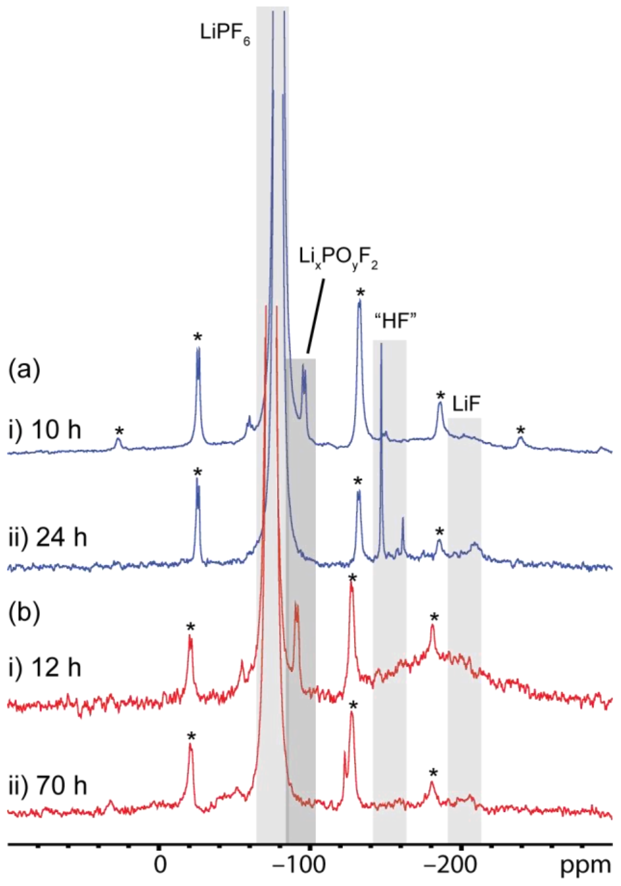

Figure S10. ^{19}F ssNMR of lithium microstructures plated on (a) c-AcH after SEI formation at 2 V in LP30 for (i) 10 hours and (ii) 24 hours (b) d-HCl-Cu after SEI formation at 2 V in LP30 for (i) 12 hours and (ii) 70 hours. Spinning side bands are marked with a star. The spectra were acquired with a MAS frequency of 25 kHz.

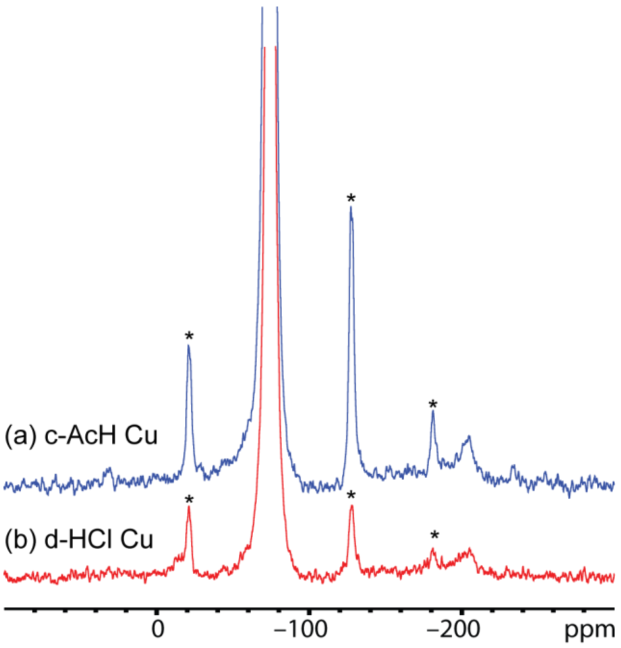

Figure S11. ^{19}F ssNMR spectra of e-SEI$_{1.4V}$ on lithium microstructures plated on (a) d-AcH-Cu after SEI formation at 1.4 V in LP30 for 80 hours and (b) d-HCl-Cu for 88 hours. Spinning side bands are marked with asterisks.

In order to establish whether the origin of the additional resonances is the Cu surface chemistry or the presence of lithium metal in the cell during the rest time, the ^{19}F NMR spectrum of Cu samples treated by both methods soaked either with or without a lithium disk in the coin cell were measured (Figure S12). The resonances were observed for d-HCl-Cu soaked in electrolyte regardless of the soaking time or presence of lithium metal (Figures 5, S11, S12). Thus, these resonances could not be solely correlated to the treatment of the Cu or to the presence of lithium metal.

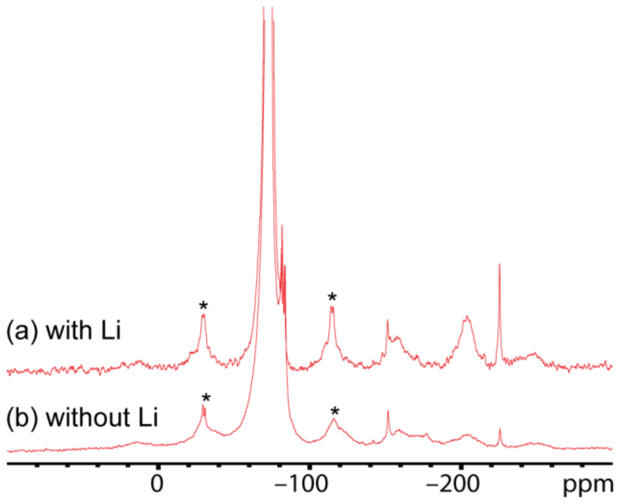

Figure S12. ^{19}F ssNMR spectra of N-SEI Cu-d-HCl soaked in LP30 for 96 hours (a) with lithium and (b) without lithium. Spinning side bands are marked with asterisks.

^{19}F NMR resonances in the range of -153 ppm to -159 ppm were assigned to HF. However, it is not fully clear how HF could exist in the solid phase and co-exist with Li metal (for the Li metal containing samples). A possible explanation could be that the origin for the HF signal is the hydrogen bond between OH^- ions and LiF on the Cu surface.[13–17]

XPS

The surface of d-HCl-Cu and c-AcH-Cu was studied by XPS. The d-HCl-Cu is surface is composed of Cu metal and Cu(I), while c-AcH-Cu is characterized by a mix of Cu(II) and Cu(I) oxides (Figures 4, S13). Since the Cu metal signal is less pronounced, the oxide coverage seems to be more homogeneous on c-AcH-Cu. Peaks at 931-932.4 eV (Cu 2p3/2) and 951.9-952.2 eV (Cu 2p1/2), observed in the Cu 2p spectrum and the O 1s peak at 530.2 eV are assigned to Cu metal and Cu(I) rich copper oxides, respectively (Figures 4, S13). The shoulder observed in the Cu 2p spectrum of c-AcH-Cu and the pristine copper current collector at 934-935 eV, the minor shake-up feature at 941-945 eV and the 531-531.2 eV peaks in the O1s spectrum serve as an indication for the presence of Cu(II) compounds (Figures 4, S13). The SEI formed on the surface of d-HCl-Cu was chosen to be studied since d-HCl pre-treatment is a typical preparation procedure for copper current collectors in the literature.

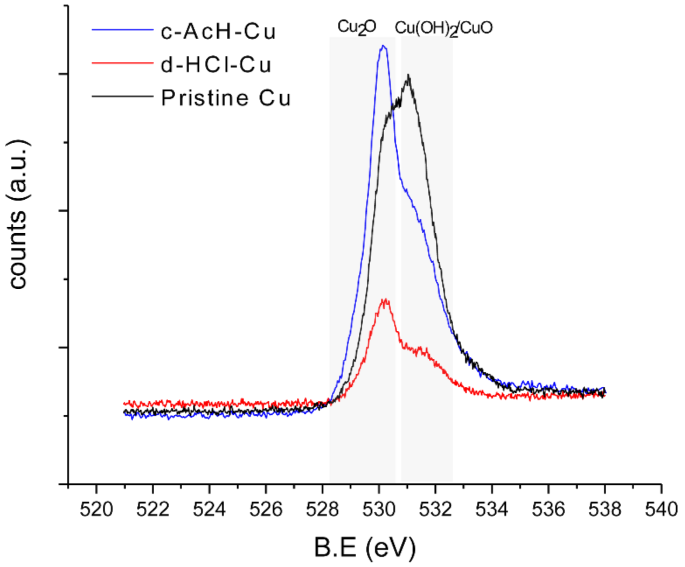

Figure S13. O 1s XPS spectrum of pristine (black), HCl treated (red) and acetic acid treated (blue) copper current collectors.

The peaks at 932 eV ($Cu2p_{3/2}$) and 951.75 eV ($Cu2p_{1/2}$) were observed in the Cu 2p XPS spectra of N-SEI, e-SEI_{2V} and d-HCl-Cu foil (Figure S14). These peaks are typical to the spectrum of Cu metal and Cu_2O. In addition, a slight shoulder at 934-935 eV was observed for e-SEI_{2V}, it can be assigned to $Cu(OH)_2$. The minor shake-up feature at 941-945 eV, indicating the presence of Cu(II) compounds, suggests further reactivity of the Cu compounds during SEI formation around 2V.

The Cu 2p spectrum for $eSEI_{1.4V}$ (Figure S15) is significantly different; no Cu 2p peaks were observed measuring the surface of this sample. After 10 nm sputtering peaks at 932.0 eV (Cu $2p_{3/2}$) and 952 eV (Cu $2p_{1/2}$) appeared. This spectrum is very similar to that recorded for the N-SEI and $eSEI_{2V}$ and could suggest that applying 1.4 V constant voltage gives rise to a thicker SEI. Further sputtering of an additional 10 nm gives rise to negative shift of both peaks (931.5, 951.35 eV) indicating possible Cu oxide lithiation or the formation of Cu nanoparticles. [18,19]

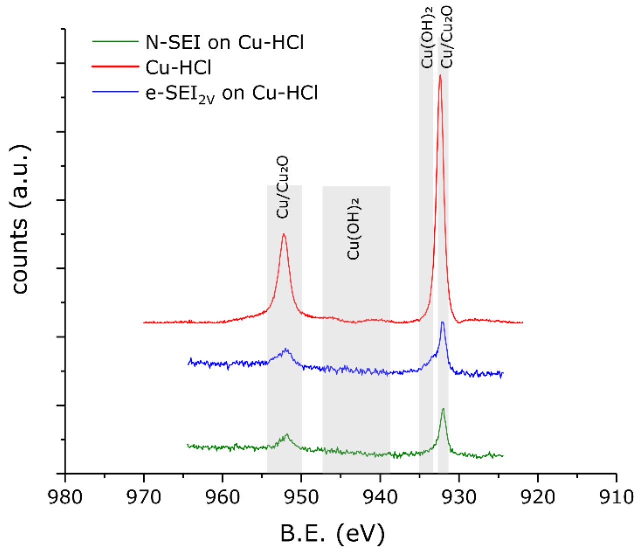

Figure S14. Cu 2p XPS spectrum of d-HCl-Cu (red), N-SEI on d-HCl-Cu (green) and eSEI$_{2V}$ d-HCl-Cu (blue).

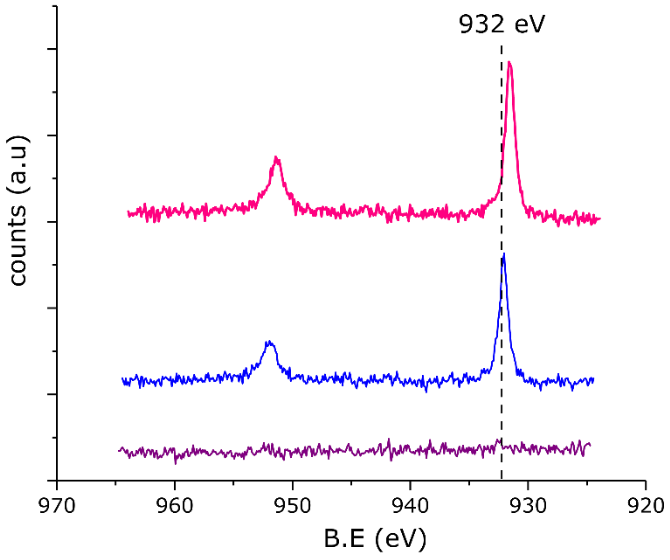

Figure S15. Cu 2p XPS spectrum of eSEI$_{1.4V}$ on d-HCl-Cu surface (purple), 10 nm sputtering (blue) and 20 nm sputtering (pink). The black line is placed for visual reference to the binding energy shift upon sputtering.

The C 1s spectrum of N-SEI includes peaks at 286.4 eV (C-O) and 288.7 eV (C=O) as a result of solidified ethylene carbonate (Figure S16a). The corresponding O 1s spectrum is deconvoluted to two peaks at 532 eV (C=O) and 533.4 (C-O) (Figure S16b). Since EC

solidification occurred during sample preparation, it should not be considered as a component of the N-SEI. The O 1s peak around 532 eV could also have a contribution from oxygen atoms in Cu oxides and phosphates (P-O) which result from the decomposition of the $LiPF_6$ salt. The presence of phosphates is further supported by the P 2p signal around 132-140 eV assigned to $Li_xPO_yF_z$ (Figure S17). [20] The broad lithium peak around 56 eV indicates the presence of $LiPF_6$ along with its decomposition products (Li_xPF_y, $Li_xPO_yF_z$) and LiF (Figure S16d) which is also in corroboration with the presence of F1s peaks at 685.3 eV (typical for LiF) and 687.2 eV (typical for $LiPF_6$ decomposition products) (Figure S16c). [21] These findings suggest that the N-SEI on Cu is composed of Cu oxides and the $LiPF_6$ decomposition products also seen in the ^{19}F NMR.

The XPS C 1s, O 1s, F1s and Li1s spectra of e-SEI_{2V} are very similar to the spectra recorded for the N-SEI (Figure S16). However, the C1s spectrum shows an increase in the intensity of C-O and C=O components in comparison to C-C/C-H components (Figure S16). In addition, the increase in the intensity of the P 2p peak (Figure S17) at 135 eV suggests the simultaneous formation of phosphates [20] and polymeric phosphorous containing PEO-like compounds at 2 V. [22]

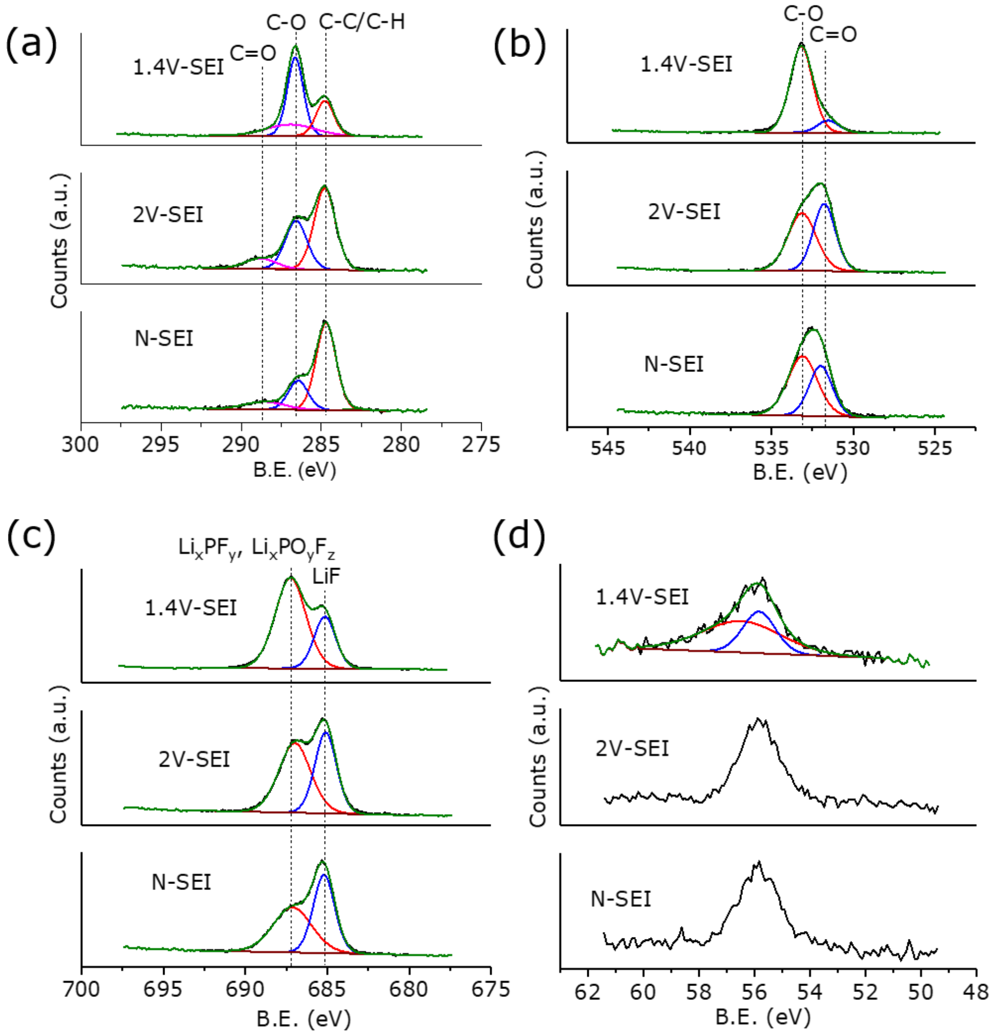

Figure S16. (a) C 1s (b) O 1s (c) F 1s and (d) Li 1s XPS spectra of N-SEI (bottom), $eSEI_{2V}$ (middle) and $eSEI_{1.4\ V}$ (top). The recorded and final resultant fitted curves are plotted in black and green colours respectively. The components spectra are shown in blue, red and magenta colours.

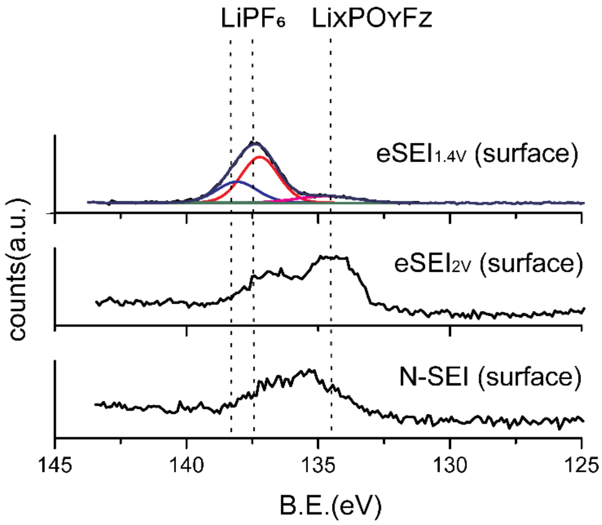

Figure S17. P 2p spectrum of N-SEI (bottom), $eSEI_{2V}$ (middle) and $eSEI_{1.4V}$ (top). The recorded and final fitted curves are plotted in black and green colours respectively. The components spectra are shown in blue, red and magenta colours.

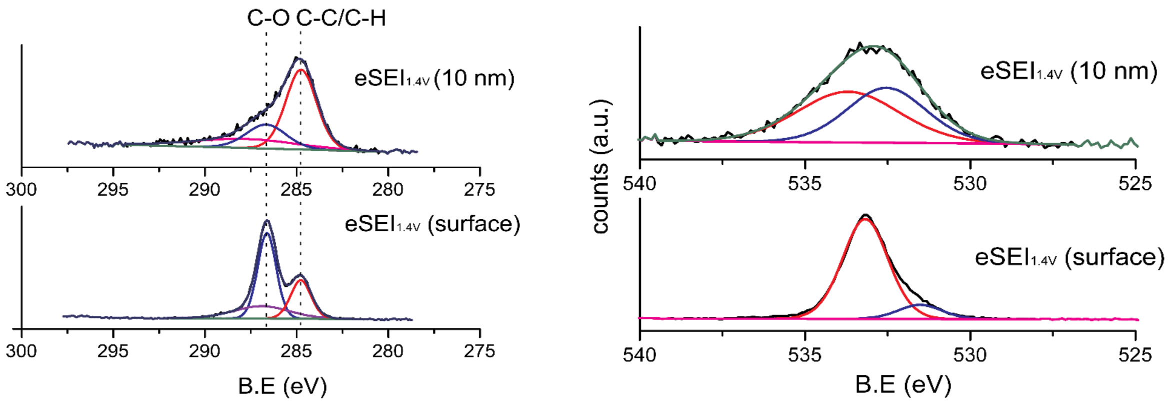

Figure S18. C 1s (left) and (b) O 1s (right) XPS spectrum of 1.4 V-SEI before and after 10 nm sputtering.

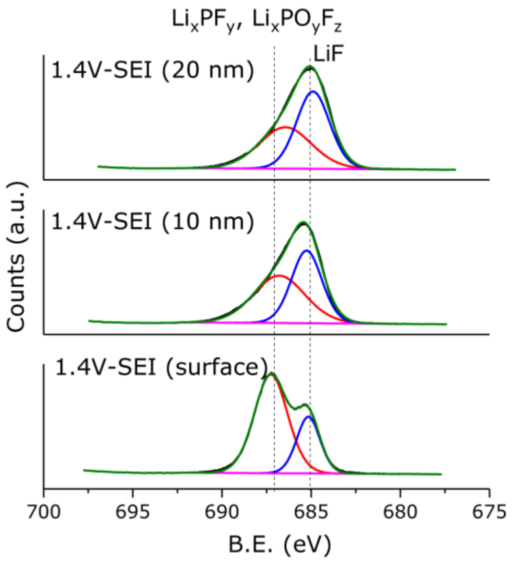

Figure S19. F 1s XPS spectrum of 1.4 V-SEI before and after 10 and 20 nm sputtering.

Table S2. Florine containing components composition as function of sputtering depth in e-SEI$_{1.4V}$

	Li_xPF_y or Li_XPO_yF (%)	LiF (%)	LiF / Li_xPF_y or Li_XPO_yF ratio
Surface	71.3	28.7	0.4
10 nm	52.5	47.5	0.9
20 nm	45.7	54.3	1.18

The composition of the SEI on lithium plated Cu substrates was studied with XPS both on the plated lithium and on the surface of the exposed Cu SEI areas (Figures S20-21). Similarly to the e-SEI$_{1.4V}$ (Figure S15), the Cu 2p signals were not observed on the surface, presumably since the SEI on the lithium plated sample is thicker and the Cu compounds are buried deeper than 20 nm (Figure S20). After sputtering 20 nm of the exposed Cu area, the Cu 2p spectrum was observed with more significant negative shifts (929.7 eV and 949.0 eV), this could be due

to both or either Cu oxide lithiation and Cu metal nano-cluster formation. [18–20,23] Sputtering of 20 nm of the lithium-plated area did not give rise to any Cu 2p peaks (Figure S20).

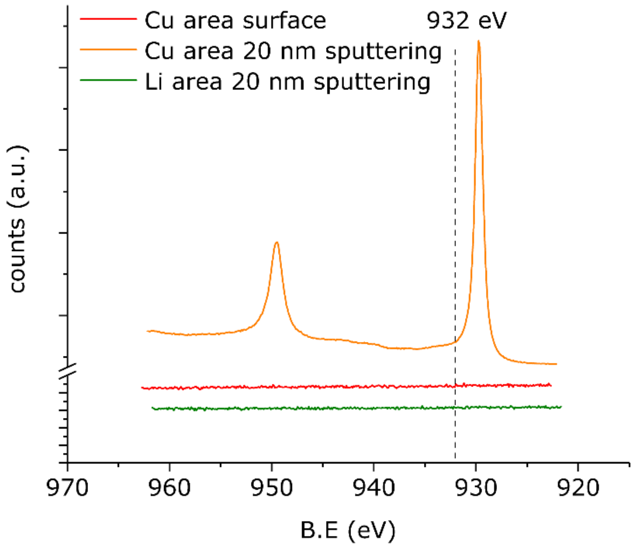

Figure S20. Cu 2p XPS spectrum of SEI on lithium plated d-HCl-Cu surface (non-plated area) (red), 20 nm sputtering (orange) and 20 nm sputtered lithium plated area (green).

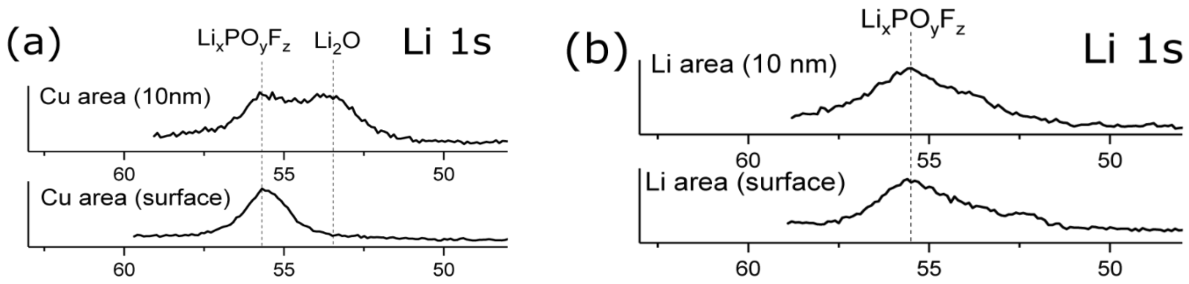

Figure S21. Li 1s XPS spectra of SEI on Li (a) and Cu (b) areas on plated d-HCl-Cu area.

***In situ* NMR**

The skin depth of Li metal in this study is $d = \sqrt{\frac{\rho}{\pi \mu_0 \mu_r \nu}} = 12.1$ µm where ρ is the resistivity of the metal (94.7 n Ω for Li metal at 298 K), μ_0 is the permeability of the vacuum (4π 10^{-7} m kg/ s^2A^2), μ_r is the relative permeability of the medium ($\mu_r = 1.4$ for Li metal) and ν is the frequency of the applied rf field (116.7 MHz).[24,25]

As shown in our previous work using in situ NMR on Cu-LFP full cells,[26] skin depth issues are not expected to be an issue. Similar to the previous study, only 1 mAh cm^{-2} of Li metal is plated in each cycle and by performing a "nutation experiment" of plated Li, we showed that the electrodeposits nutated like a sample experiencing no skin depth effects. Furthermore, the changes in Li metal intensity (in Figure 7) are approximately linear with charge, indicating that all plated Li contributes to the NMR signal.

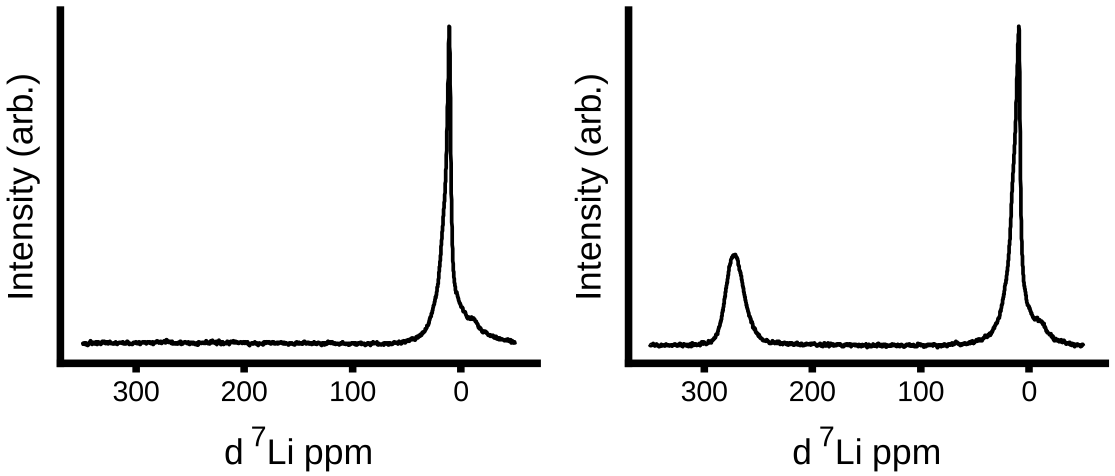

Figure S22. a) The ^{7}Li in situ NMR spectra at the start of cycling where only the diamagnetic peak originating from the electrolyte is observed. b) The ^{7}Li in situ NMR spectra at the end of charge (1 mAh/cm^2) showing both the Li metal peak centered around approximately 260 ppm and the electrolyte peak.

The coulombic efficiency (CE) of the galvanostatic cycling performed in in situ NMR cells and the corresponding normalized intensity of the Li metal peak were quantified with in situ NMR at the end of charge (plating) and discharge (stripping) for d-HCl-Cu (Figure S23) and c-AcH-Cu (Figure S24).These are additional examples of the trends described in the manuscript. The CE and the amount of dead Li quantified with NMR (the intensity of Li metal at the end of discharge) is similar for both surface treatments whereas the intensity of Li metal at the end of charge is different for the two cells.

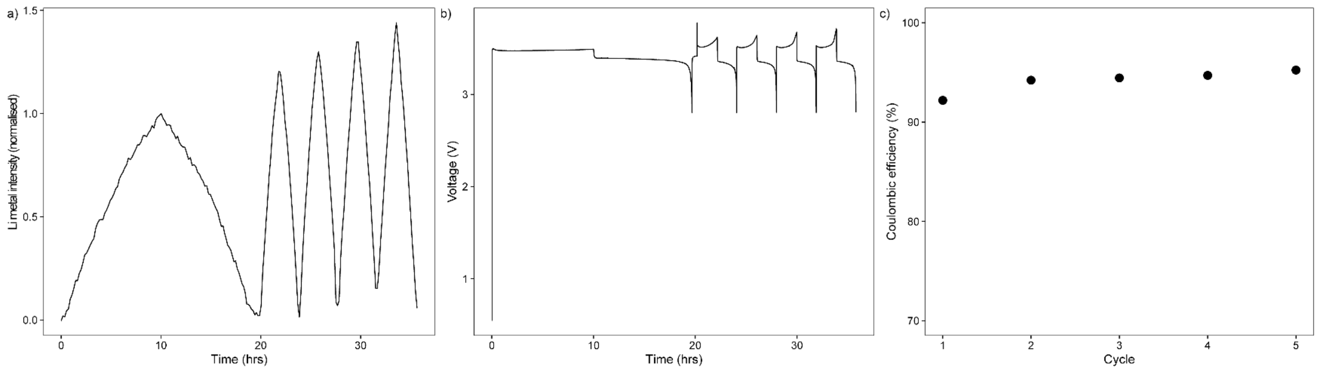

Figure S23. a) Lithium metal intensity plot b) Galvanostatic cycling c) CE of in situ Cu-LFP cells with d-HCl-Cu where 0.1 mA/cm^2 was used for the first cycle and 0.5 mA/cm^2 for the subsequent cycles. The equivalent charge of 1 mAh/cm^2 was passed.

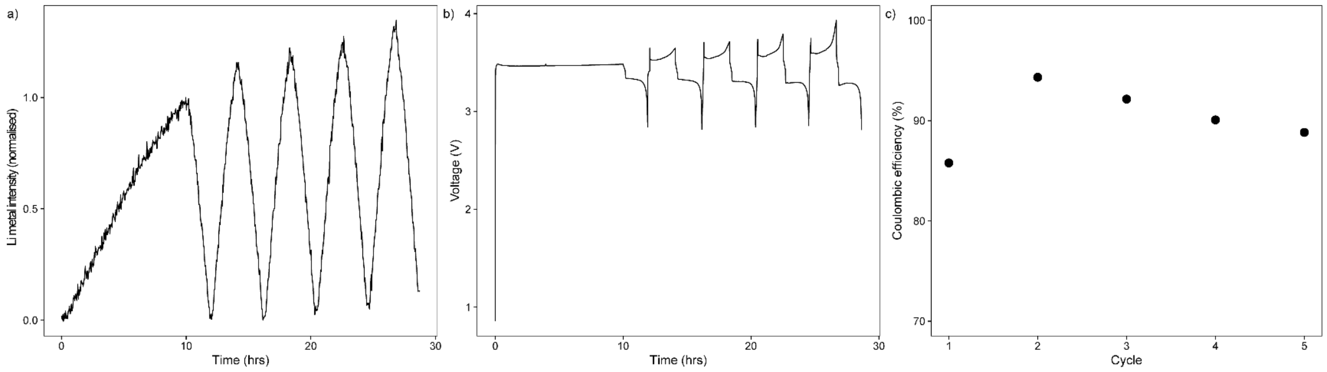

Figure S24. a) Lithium metal intensity plot b) Galvanostatic cycling c) CE of in situ Cu-LFP cells with c-AcH-Cu where 0.1 mA/cm^2 was used for the first plating and 0.5 mA/cm^2 for the subsequent cycles. The equivalent charge of 1 mAh/cm^2 was passed.

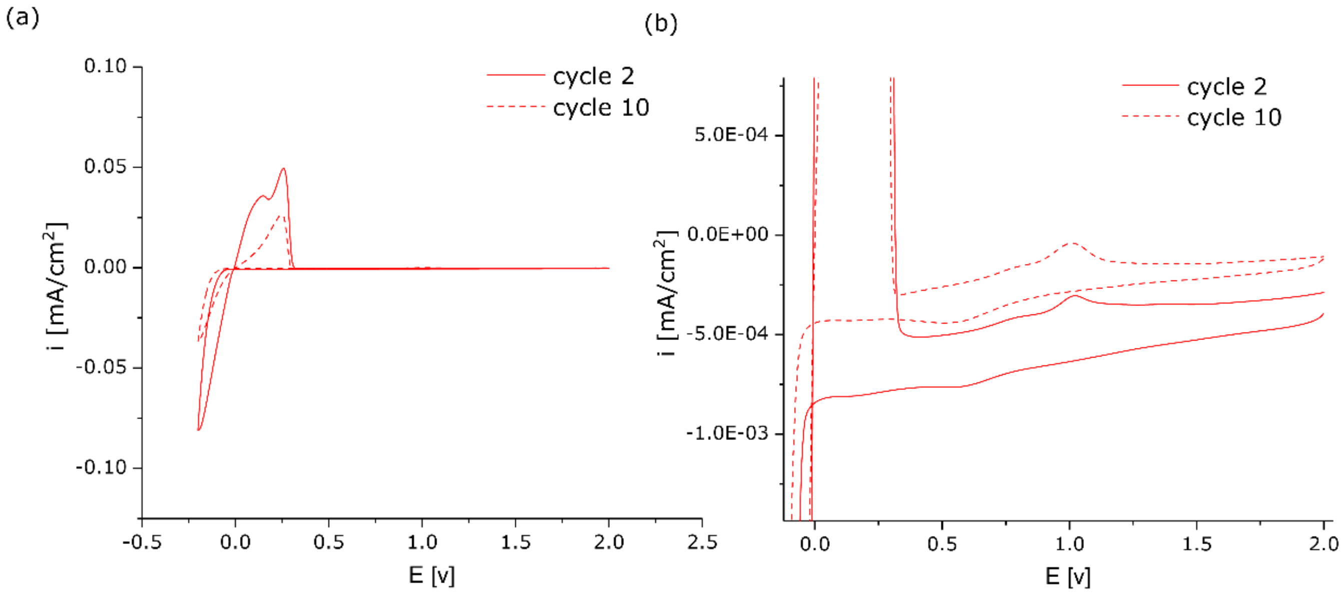

Figure S25. (a)2nd (full line) and 10th (dashed line) CV cycle of lithium plating on d-HCl-Cu in three-electrode cell, Cu WE vs. Li disk CE and RE, with scan rate 1mV/sec (b) magnification of the semi-reversible reaction peaks.

TOF-SIMS

The measurements were conducted in the burst alignment mode (BAM) for better lateral resolution of images with a Bi^+ primary beam (25 keV) and a Cs^+ sputtering beam (500 eV) over an area of 250 μm × 250 μm (sputtering area 500 μm × 500 μm). During the measurements, the current for the sputtering beam was steady and constant (35 nA), thus over the same sputtering span, the fluence dose density were expected to be the same for all the samples. Moreover, the high current bunch mode (HCBM) with a mass resolution up to 10000 was also applied to ensure that within the limitation of the instrument, the species analysed in BAM mode were solely contributing. For these air sensitive samples, they were mounted inside an Ar-filled glovebox and transferred into the instrument within the vacuum transport suitcase which was opened until the pressure of the loadlock chamber lower than 10^{-5} mbar.

The intensity of different species in SIMS are affected by a variety of different factors and the concentration of species normally cannot be obtained directly from the intensity data. However, for the same species, assuming the chemical environment was similar within the same sample and among samples with similar treatments, the relative intensities correlated with related concentrations are comparable.

Native and electrochemical SEI on Cu were studied by ToF-SIMS. Cu_xO, LiF, CuF_x and OH^- -related species were observed in N-SEI and e-SEI at all tested conditions. Since the penetration depth of the SIMS is on the order of the thickness of N-SEI and constant-voltage e-SEI, the acquired depth profiles were analysed qualitatively.

In Figure S26 the depth profiles of the copper metal (detected as Cu_2^-) on N-SEI or e-SEI are depicted. The sharp increase of the copper metal signal (detected as Cu_2^-) served as an indication of the end of SEI sputtering (Figure S26). The thickness of the N-SEI is lesser than the penetration depth of the ToF-SIMS experiment, thus the copper metal intensity increases immediately at the start of sputtering, while the e-SEI is thicker and as a result the intensity increase occurs after a lag. The sharp increase in the Cu metal signal occurs after longer sputtering for the N-SEI and e-SEIs on c-AcH-Cu, thus the SEIs formed on c-AcH-Cu are thicker compared to the ones on d-HCl-Cu.

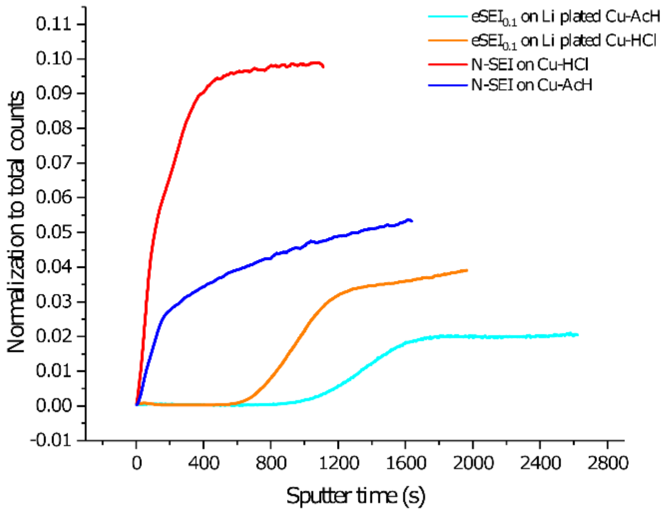

Figure S26. Cu metal depth profile of N-SEI and $eSEI_{0.1V}$ on lithium plated copper

Lithium fluoride (LiF, detected as LiF_2^-) depth profiles on d-HCl-Cu are presented in Figure S27a and S27c. The LiF intensity is significantly higher for the high voltages (2 and 2.8 V) compared to N-SEI (rest) and the low voltage steps (1.4 V and 0.1 V). This suggests that most of the LiF forms at high voltages (i.e., 2 and 2.8 V). The LiF depth profile in N-SEI on d-HCl-Cu vs c-AcH-Cu (Figure S27c) indicates thicker N-SEI on c-AcH-Cu.

The depth profiles of $^{65}CuO^-$ in N-SEI on d-HCl-Cu and c-AcH-Cu are depicted in Figure S27b. The intensity of $^{65}CuO^-$ is significantly higher for d-HCl-Cu samples. This could be an indication of a thicker or more homogeneous SEI on c-AcH-Cu.

Copper fluorides (detected as $^{65}CuF_2^-$) were observed in N-SEI and SEI on copper by TOF-SIMS (Figure S27d). The $^{65}CuF_2^-$ depth profile of N-SEI and SEI reveals similar behaviour to copper oxides. The intensity of $^{65}CuF_2^-$ is higher for N-SEI and $eSEI_{2.8}$ compared to eSEIs formed at lower voltages. This suggests that CuF_x are found mainly in inner SEI layers. Moreover, CuF_2 was observed in the XPS spectra. F1s gives rise to a broad peak for CuF_2 around 685 eV, however this peak overlaps with LiF. Copper fluorides were not found by ssNMR, this is probably because CuF_2 contains paramagnetic Cu(II) that is not visible to ssNMR.

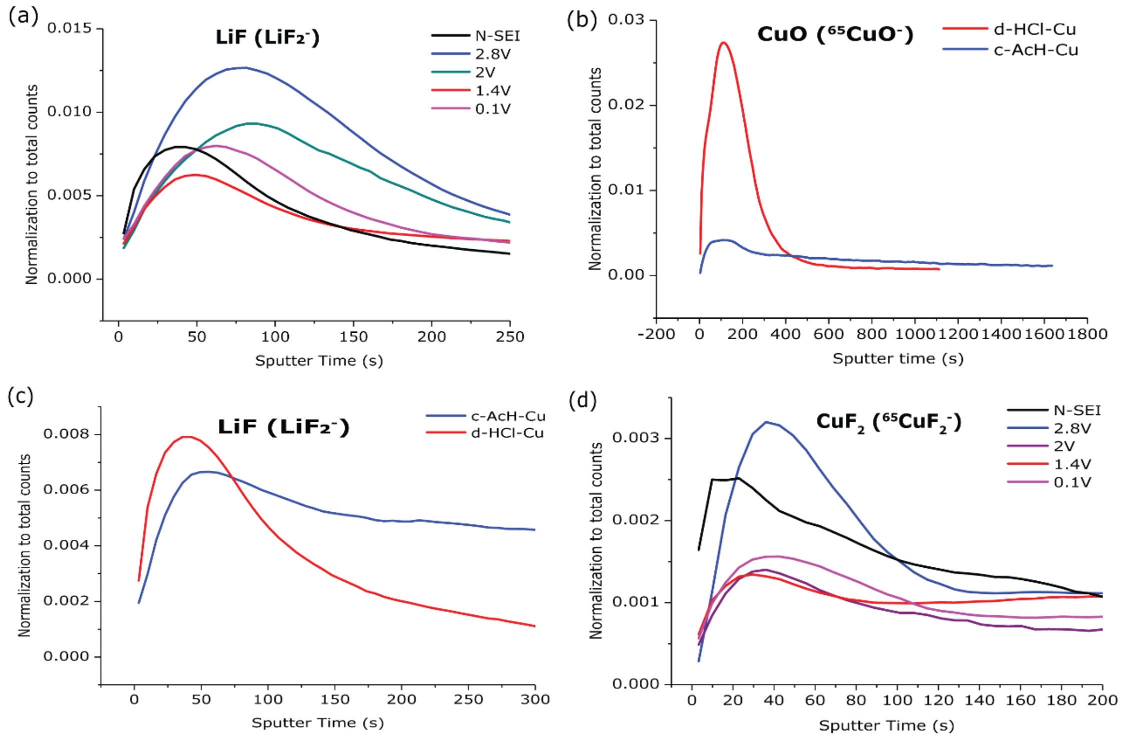

Figure S27. (a) LiF (LiF$_2^-$) depth profile in constant-voltage and N-SEI on d-HCl-Cu. (b) ^{65}CuO depth profile in N-SEI on d-HCl-Cu and c-AcH-Cu. (c) LiF depth profile in N-SEI on d-HCl-Cu (red) and c-AcH-Cu (blue). (d)CuF$_2$ (^{65}CuF$_2$) depth profile in e-SEI formed at 0.1,1.4,2,2.8 V on d-Cu-HCl. The presented profiles were limited to the sputtering time at which the Cu metal signal intensity increased abruptly.

The presented ToFSIMS depth profiles are the results of signal collection from the analysis area (250 μm × 250 μm). However, further data analysis of the ^{65}CuO signal from different regions of interest, i.e., the exposed copper and plated lithium, confirmed the presence of copper oxides in the SEI on both areas. The calculated thickness of the plated lithium is approximately 1.5 μm assuming 100% efficiency and coverage of 50% of the copper area. Thus, it is assumed that for all the SIMS analysis, the copper oxides remaining on the underlying copper substrates cannot contribute to the copper oxide signals recorded from the lithium surface.

The 3D reconstruction of $^{65}CuO^-$ signal (XZ plane) of $eSEI_{0.1V}$ on lithium platted d-HCl-Cu (right) and c-AcH-Cu (left) in depicted in Figure S28. For each sample the $^{65}CuO^-$ signals for SEI on lithium and the exposed Cu are compared. The intensity of $^{65}CuO^-$ in the SEI on the exposed d-HCl-Cu is higher compared to the exposed c-AcH-Cu and that the increase of $^{65}CuO^-$ signal with sputtering is more gradual on d-HCl-Cu. The intensity of the $^{65}CuO^-$ signal from the SEI on the plated lithium is similar to the signal from the top layers of he SEI on the exposed copper.

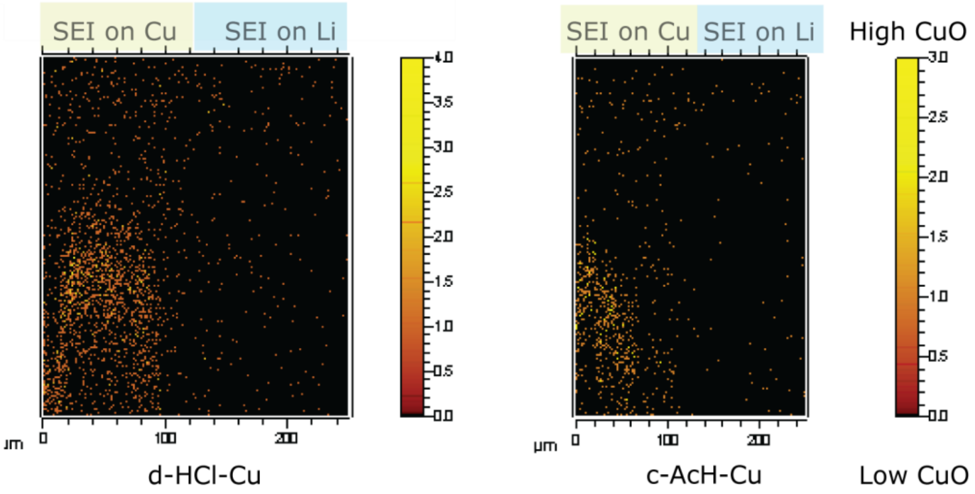

Figure S28. 3D reconstruction of ^{65}CuO signal (XZ plane), comparing the intensity of ^{65}CuO on exposed and lithium plated areas on a copper disk after the application of lithium plating procedure (d-HCl-Cu-right, c-AcH-Cu- left).

Lithium plating on Cu

The XY maps of OH^- and LiF (detected as LiF_2^-) in the eSEI$_{0.1v}$ on c-AcH-Cu (Figure S29), LiF and OH^- XY and XZ maps of eSEI$_{2V}$ on c-AcH-Cu (Figure S30) and d-HCl-Cu (Figure S31) reveal a similar trend to the SEI composition trend on d-HCl-Cu (Figure 10). In Figures S29-S33 the areas with the higher intensity are SEI on Cu areas.

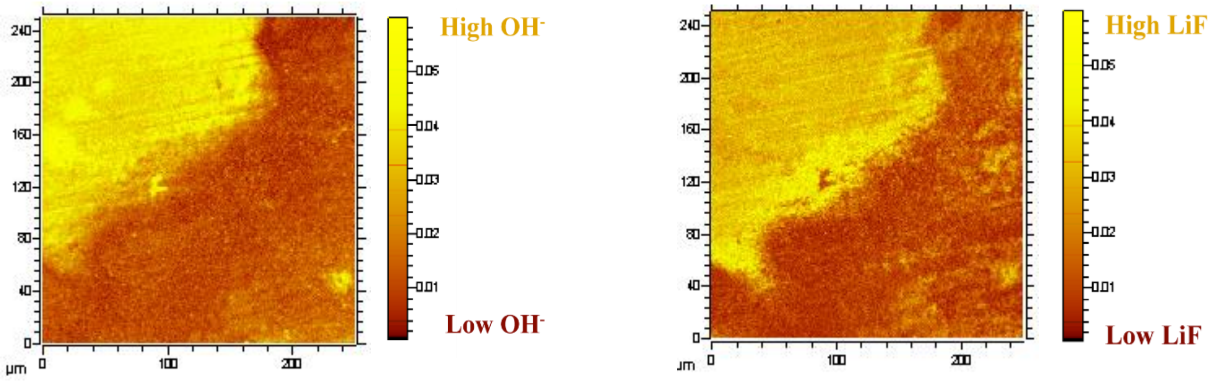

(a) (b)

Figure S29. XY (total) OH^- (a) and LiF (LiF_2^-) (b) maps of eSEI$_{0.1V}$ on c-AcH-Cu.

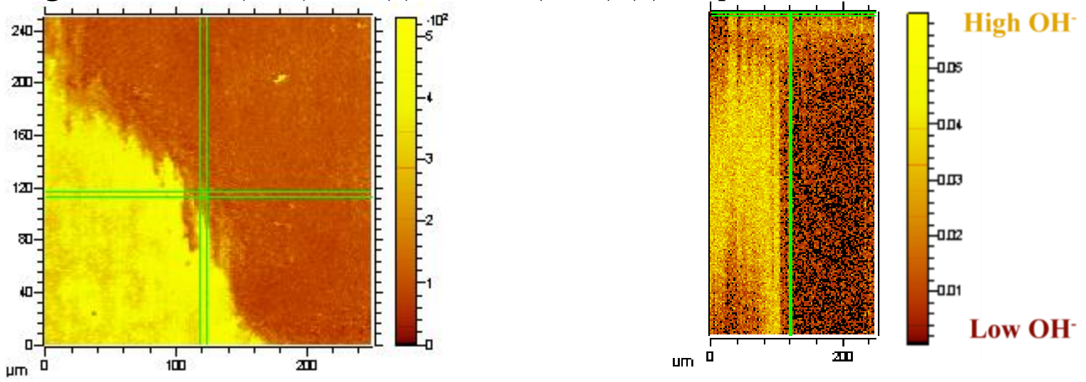

Figure S30. XY (total) (a) and XZ (5 layers) (b) maps of OH^- on eSEI$_{2V}$ on c-AcH-Cu, recorded on both lithium-plated and bare regions with equal sizes areas of 50X50 µm.

(a) (b)

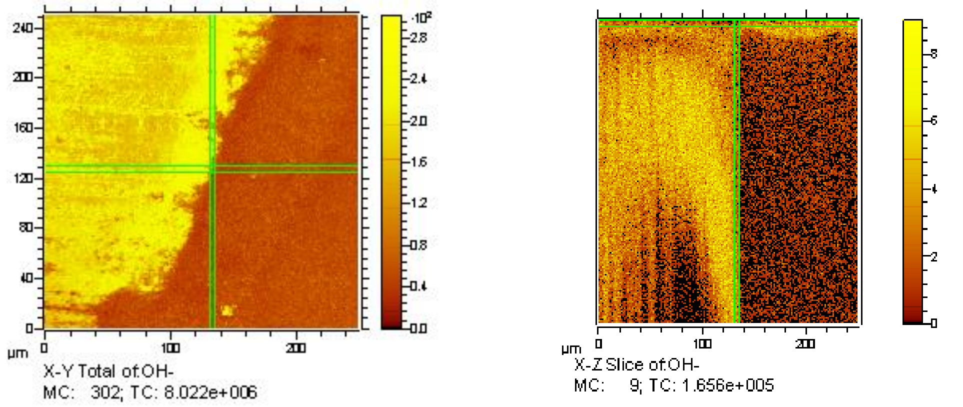

Figure S31. XY (total) (a) and XZ (5 layers) (b) maps of OH^- on $eSEI_{2V}$ on d-HCl-Cu, recorded on both lithium-plated and bare regions with equal sizes areas of 50X50 μm.

(a) (b)

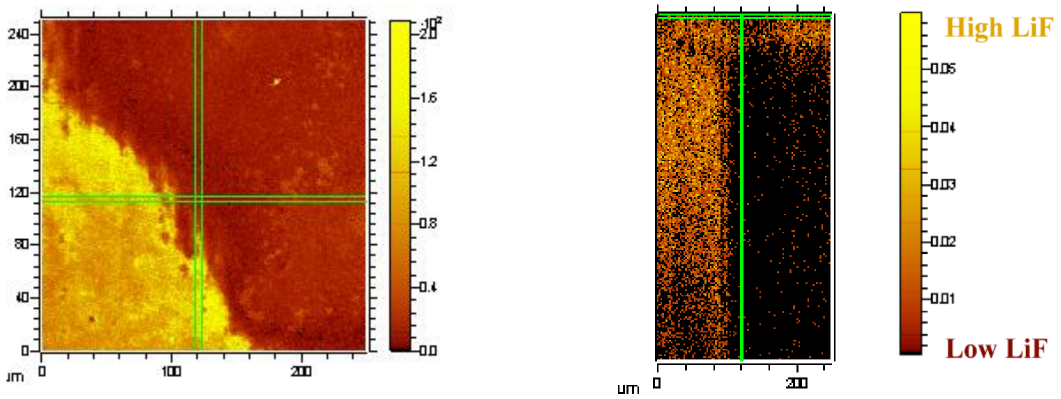

Figure S32. XY (total) (a) and XZ (5 layers) (b) maps of LiF (LiF_2^-) on $eSEI_{2V}$ on c-AcH-Cu.

(a) (b)

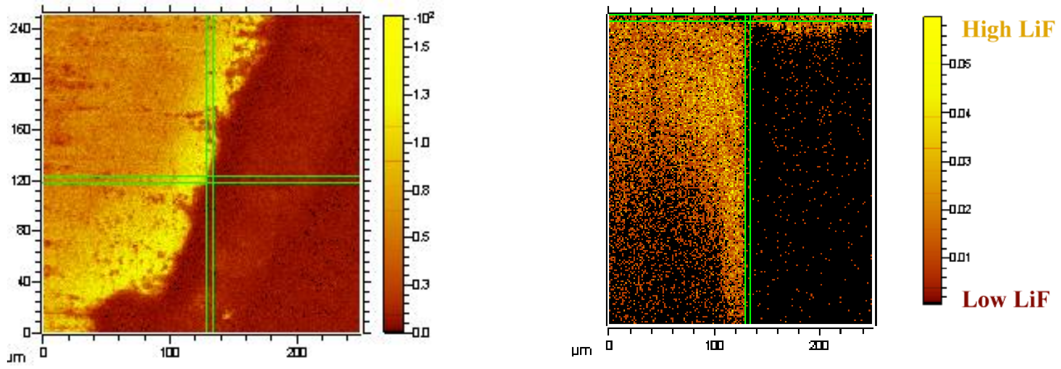

Figure S33. XY (total) (a) and XZ (5 layers) (b) maps of LiF (LiF_2^-) on $eSEI_{2V}$ on d-HCl-Cu. OH^- depth profiles of N-SEI on d-HCl-Cu and c-AcH-Cu samples are shown in Figure S34.

The OH^- intensity in N-SEI on d-HCl-Cu is higher. The intensity of the OH^- signal for the N-SEI on d-HCl-Cu is significantly higher and more heterogeneous compared to that of N-SEI on c-AcH-Cu. These findings strengthen the reliability of the OH^- mapping and support the assumption of preferential Li plating on OH^- poor areas.

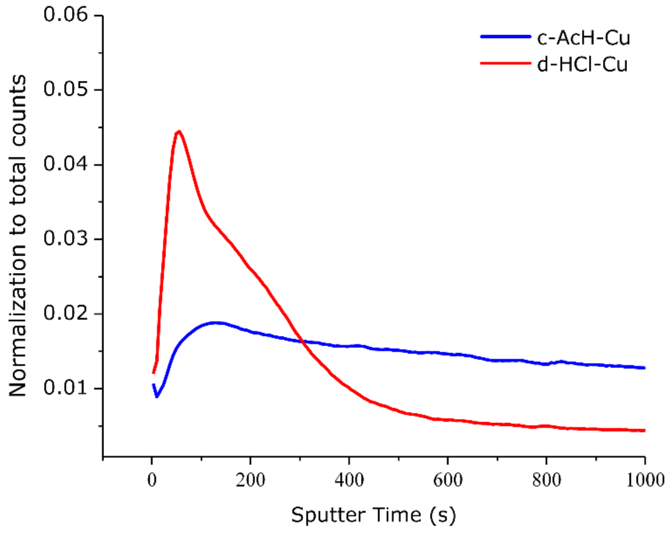

Figure S34. OH^- depth profile in SEI formed at N-SEI, normalised to total counts

	Energy [eV]	Assignment	Reference
C 1s	282.15	Li_2C_2	[21]
	284.4-284.8	C-C, C-H	[21]
	286.4-286.6	C-O-C, PEO, $(CH_2OCO_2Li)_2$	
	288.7-288.8	C=O	
	288.8-289.3	CO_3^-	
O 1s	528	Li_2O	[21]
	531	LiOH or H_2O_{ad}	[27,21]
	531.2	P=O or Li_2O_2	
	531.7	LiOH or H_2O_{ad}	
	532-532.6	P-O or C=O	[28]
	533.5	PEO, C-O	[21]
Cu 2p	929.7-931.5, 949-951.35	Li_xCuO or Cu np	[29,30,20,28,31]
Cu 2p	932, 951.75	Cu, Cu(I)	
Cu 2p	934-935	Cu (II) in $Cu(OH)_2$	
Li 1s	53-54	Li_2O	
	56	Li_xPFy, $Li_xPO_yF_z$	[20,21,32]
F 1s	685.3	LiF	[21]
F 1s	687.2-687.3	$LiPF_6$	[21]
P 2p	135	Phosphates	

Table S2. C1s, O1s, F1s, Li1s, P2p and Cu2p peak energies used for the XPS analysis

**Compound**	**Detected as**	**Mass**
LiF	$Li_2F_3^-$	71.0280
LiF	LiF_2^-	45.0132
Cu_xO	$^{65}CuO^-$	80.9233
Cu metal	Cu_2^-	125.8598
OH^-	OH^-	17.0033

Table S3. main compounds observed in TOFSIMS analysis.

References

1. Balbuena, P. B. *Lithium-lon Batteries Solid-Electrolyte Interphase*. (Imperial College Press, 2004).

2. Irvine, B. J. T. S., Sinclair, D. C. & West, A. R. <Electroceramics Characterisation by impedance sepctroscopy.pdf>. **2**, 132–138 (1990).

3. Ross Macdonald, E. B. *Fundamentals of Impedance Spectroscopy*.

4. Eshkenazy, V. THE ROLE OF SEI IN LITHIUM AND LITHIUM ION BATTERIES E. PELED, D. GOLODNITSKY, G. ARDEL, C. MENACHEM, D. BAR TOW AND V. ESHKENAZY School of Chemistry, Tel Aviv University, Tel Aviv 69978, Israel. **393**, 209–221 (2020).

5. Diard, Jean-Paul & Le Gorrec, Bernard & Montella, C. *Handbook of Electrochemical Impedance Spectroscopy CIRCUITS made of RESISTORS and CAPACITORS.* (2007). doi:10.13140/RG.2.2.25393.12641.

6. IRVINE, J. T. S., SINCLAIR, D. C. & WEST, A. R. ChemInform Abstract: Electroceramics: Characterization by Impedance Spectroscopy. *ChemInform* **21**, 132–138 (2016).

7. Campion, C. L., Li, W. & Lucht, B. L. Thermal Decomposition of LiPF 6 -Based Electrolytes for Lithium-Ion Batteries. *J. Electrochem. Soc.* **152**, A2327–A2334 (2005).

8. Co, L. *et al.* Surface Chemistry Dependence on Aluminum Doping in Ni-rich. 1–12 (2019) doi:10.1038/s41598-019-53932-6.

9. Grey, C. P. & Corbin, D. R. 19F and 27Al MAS NMR study of the dehydrofluorination reaction of hydrofluorocarbon-134 over basic faujasite zeolites. *J. Phys. Chem.* **99**, 16821–16823 (1995).

10. Scholz, G., Stosiek, C., Noack, J. & Kemnitz, E. Local fluorine environments in nanoscopic magnesium hydr(oxide) fluorides studied by 19F MAS NMR. *J. Fluor. Chem.* **132**, 1079–1085 (2011).

11. Meyer, B. M., Leifer, N., Sakamoto, S., Greenbaum, S. G. & Grey, C. P. High Field Multinuclear NMR Investigation of the SEI Layer in Lithium Rechargeable Batteries. 145–148 (2005) doi:10.1149/1.1854117.

12. Huff, L. A. *et al.* Identification of Li-Ion Battery SEI Compounds through 7Li and 13C Solid-State MAS NMR Spectroscopy and MALDI-TOF Mass Spectrometry. *ACS Appl. Mater. Interfaces* **8**, 371–380 (2016).

13. Wiemers-Meyer, S., Winter, M. & Nowak, S. Mechanistic insights into lithium ion battery electrolyte degradation-a quantitative NMR study. *Phys. Chem. Chem. Phys.* **18**, 26595–26601 (2016).

14. Shenderovich, I. G. *et al.* Low-temperature NMR studies of the structure and dynamics of a novel series of acid-base complexes of HF with collidine exhibiting scalar

couplings across hydrogen bonds. *J. Am. Chem. Soc.* **125**, 11710–11720 (2003).

15. Clément, R. J., Kitchaev, D., Lee, J. & Gerbrand Ceder. Short-Range Order and Unusual Modes of Nickel Redox in a Fluorine-Substituted Disordered Rocksalt Oxide Lithium-Ion Cathode. *Chem. Mater.* **30**, 6945–6956 (2018).

16. Magnetic, N. & Studies, R. of Ion. 1878–1883 (1977).

17. T.G.Stoebe. Influence of OH- ions on infrared absorption and ionic conductivity in lithium fluoride crystals. *J. Phys. Chnn. Solid Fergamon* **28**, 1375–1382 (1967).

18. Burkstrand, J. M. Unusual core level spectra of copper on polystyrene. *Surf. Sci.* **78**, 513–517 (1978).

19. Zatsepin, D. A. *et al.* Valence states of copper ions and electronic structure of LiCu2O2. *Phys. Rev. B* **57**, 4377–4381 (1998).

20. Martin, L., Martinez, H., Poinot, D., Pecquenard, B. & Le Cras, F. Comprehensive X-ray photoelectron spectroscopy study of the conversion reaction mechanism of CuO in lithiated thin film electrodes. *J. Phys. Chem. C* **117**, 4421–4430 (2013).

21. Eshetu, G. G. *et al.* In-Depth Interfacial Chemistry and Reactivity Focused Investigation of Lithium-Imide- and Lithium-Imidazole-Based Electrolytes. *ACS Appl. Mater. Interfaces* **8**, 16087–16100 (2016).

22. Campion, C. L., Li, W. & Lucht, B. L. Thermal decomposition of LiPF 6-based electrolytes for lithium-ion batteries. *J. Electrochem. Soc.* **152**, 2327–2334 (2005).

23. Yamakawa, N., Jiang, M. & Grey, C. P. Investigation of the conversion reaction mechanisms for binarycopper(ii) compounds by solid-state nmr spectroscopy and X-ray diffraction. *Chem. Mater.* **21**, 3162–3176 (2009).

24. Kittel, C. Introduction to Solid State Physics, 8th edition. *Wiley Sons, New York, NY* (2004).

25. Lide, D. R. CRC Handbook of Chemistry and Physics, 84th Edition, 2003-2004. *Handb. Chem. Phys.* (2003) doi:10.1136/oem.53.7.504.

26. Gunnarsdóttir, A. B., Amanchukwu, C. V., Menkin, S. & Grey, C. P. Noninvasive in Situ NMR Study of 'dead Lithium' Formation and Lithium Corrosion in Full-Cell Lithium Metal Batteries. *J. Am. Chem. Soc.* **142**, 20814–20827 (2020).

27. Yao, K. P. C. *et al.* Thermal stability of Li2O2 and Li2O for li-air batteries: In situ XRD and XPS studies. *J. Electrochem. Soc.* **160**, 824–831 (2013).

28. Martin, L., Martinez, H., Poinot, D., Pecquenard, B. & Le Cras, F. Direct observation of important morphology and composition changes at the surface of the CuO conversion material in lithium batteries. *J. Power Sources* **248**, 861–873 (2014).

29. Biesinger, M. C. Advanced analysis of copper X-ray photoelectron spectra. *Surf. Interface Anal.* **49**, 1325–1334 (2017).

30. Biesinger, M. C., Hart, B. R., Polack, R., Kobe, B. A. & Smart, R. S. C. Analysis of mineral surface chemistry in flotation separation using imaging XPS. *Miner. Eng.* **20**,

152–162 (2007).

31. Biesinger, M. C., Lau, L. W. M., Gerson, A. R. & Smart, R. S. C. Resolving surface chemical states in XPS analysis of first row transition metals, oxides and hydroxides: Sc, Ti, V, Cu and Zn. *Appl. Surf. Sci.* **257**, 887–898 (2010).

32. Tanaka, S., Taniguchi, M. & Tanigawa, H. XPS and UPS studies on electronic structure of Li2O. *J. Nucl. Mater.* **283**–**287**, 1405–1408 (2000).
